# Chronic Filarial Infection Provides Protection against Bacterial Sepsis by Functionally Reprogramming Macrophages

**DOI:** 10.1371/journal.ppat.1004616

**Published:** 2015-01-22

**Authors:** Fabian Gondorf, Afiat Berbudi, Benedikt C. Buerfent, Jesuthas Ajendra, Dominique Bloemker, Sabine Specht, David Schmidt, Anna-Lena Neumann, Laura E. Layland, Achim Hoerauf, Marc P. Hübner

**Affiliations:** 1 Institute for Medical Microbiology, Immunology and Parasitology, University Hospital of Bonn, Bonn, Germany; 2 Institute of Medical Psychology and Behavioral Immunobiology, University Hospital Essen, Essen, Germany; University of Medicine & Dentistry New Jersey, UNITED STATES

## Abstract

Helminths immunomodulate their hosts and induce a regulatory, anti-inflammatory milieu that prevents allergies and autoimmune diseases. Helminth immunomodulation may benefit sepsis outcome by preventing exacerbated inflammation and severe pathology, but the influence on bacterial clearance remains unclear. To address this, mice were chronically infected with the filarial nematode *Litomosoides sigmodontis (L.s.)* and the outcome of acute systemic inflammation caused by i.p. *Escherichia coli* injection was determined. L.s. infection significantly improved *E. coli*-induced hypothermia, bacterial clearance and sepsis survival and correlated with reduced concentrations of associated pro-inflammatory cytokines/chemokines and a less pronounced pro-inflammatory macrophage gene expression profile. Improved sepsis outcome in *L.s.*-infected animals was mediated by macrophages, but independent of the alternatively activated macrophage subset. Endosymbiotic Wolbachia bacteria that are present in most human pathogenic filariae, as well as *L.s.*, signal via TLR2 and modulate macrophage function. Here, gene expression profiles of peritoneal macrophages from *L.s.*-infected mice revealed a downregulation of genes involved in TLR signaling, and pulsing of macrophages in vitro with *L.s.* extract reduced LPS-triggered activation. Subsequent transfer improved sepsis outcome in naïve mice in a *Wolbachia*- and TLR2-dependent manner. In vivo, phagocytosis was increased in macrophages from *L.s.*-infected wild type, but not TLR2-deficient animals. In association, *L.s.* infection neither improved bacterial clearance in TLR2-deficient animals nor ameliorated *E. coli*-induced hypothermia and sepsis survival. These results indicate that chronic *L.s.* infection has a dual beneficial effect on bacterial sepsis, reducing pro-inflammatory immune responses and improving bacterial control. Thus, helminths and their antigens may not only improve the outcome of autoimmune and allergic diseases, but may also present new therapeutic approaches for acute inflammatory diseases that do not impair bacterial control.

## Author Summary

As the human immune system evolved in the presence of helminth infections, it is postulated that improved hygiene and subsequent loss of helminth infections and their immunomodulatory functions contributed to the sharp increase of autoimmune diseases and allergies over the last decades. Accordingly, helminth-induced anti-inflammatory, regulatory immune responses ameliorate allergy and autoimmune diseases and are likely to impact other immunological disorders including sepsis. Sepsis is an exacerbated, systemic inflammatory disease that occurs when pathogens cannot be locally confined and spread via the blood stream. Thus, efficient sepsis therapies should reduce excessive inflammation without impairing protective immune responses. In the present study we demonstrate that chronic filarial infection modulates macrophages to a less pro-inflammatory phenotype with improved phagocytic capacity. This immunomodulation reduces sepsis-induced inflammation and hypothermia and clears bacteria more efficiently thus improving sepsis survival. Moreover, we found that *Wolbachia*, the endosymbiotic bacteria of filariae, play a crucial role in triggering the correct macrophage response via TLR2. Thus, our observations suggest that helminths and helminth-derived antigens may not only present new treatment options for allergies and autoimmune diseases, but may also allow treatment of sepsis caused inflammation without impairing bacterial control.

## Introduction

Helminth infections typically induce type 2 immune responses which are characterized by the induction of Th2 cells, eosinophilia and elevated levels of IgE as well as IL-4, IL-5 and IL-13 [1]. To ensure long term host-parasite co-existence, helminths also suppress inflammatory immune responses and thereby limit pathology. Helminths strive to establish a hypo-responsive milieu in their hosts by inducing regulatory T cells, alternatively activated macrophages (AAM) and the release of anti-inflammatory cytokines (IL-10 and TGFβ), which impact both adaptive and innate immunity [2–4]. These forms of immunomodulation by helminths effect bystander immune responses and can improve autoimmune diseases [5–8] or allergy [9–12] while efficacy of vaccinations is reduced [13, 14]. Furthermore, several epidemiological reports and animal studies have demonstrated that helminth infections alter immune responses to unrelated pathogens [15–18].

With regard to bacterial co-infections, the impact of concurrent helminth infections remains incompletely understood [15, 16]. The above mentioned Th2 biasing and regulatory characteristics of helminth infections could, theoretically, impair the development of protective Th1 immunity in response to bacterial co-infections [19–21] and increase susceptibility as shown with models of *Mycobacterium tuberculosis* [22, 23]. However, several reports have not observed impaired immunity in experimental *Mycobacterium*-helminth co-infections and these variations in susceptibility seem to depend on the combination of helminth species and *Mycobacterium* strain [24, 25].

Similar inconsistent findings have been noted in other bacteria/helminth co-infection models as well. For example, intracellular killing of *Citrobacter rodentium* is hampered due to impaired autophagy in IL-4 receptor- / STAT6- induced AAM in *Heligmosoides polygyrus* infected mice [26]. On the other hand, pre-existing *Nippostrongylus brasiliensis* infection improves *Klebsiella pneumoniae* induced septic peritonitis in an IL-4 and mast cell dependent manner [27]. Furthermore, improved outcomes in animal models of endotoxemia were attributed to the modulation of TLR-signaling or LPS sensing; the agglutination cascade through structural components and excretory/secretory products of nematodes [28–31]. Indeed, filarial nematodes are potent modulators of the immune system which allows their persistence in the human host for more than a decade. Interestingly, most human pathogenic filarial nematodes contain endosymbiotic *Wolbachia* bacteria that are essential for filarial development. *Wolbachia* have the capacity to trigger pro-inflammatory responses [32, 33] and have therefore been implemented in the development of pathology during filarial infection [34].

Thus, filariae (and their endosymbionts) can modulate the immune system of their hosts which affects their immune responses to co-infections. Currently, there is a working hypothesis that impairment of protective immune responses by helminths may facilitate the dissemination of pathogens via the blood stream and cause sepsis. Severely dysregulated immune responses during sepsis initially lead to a systemic inflammatory response syndrome (SIRS) which may result in tissue damage, organ failure and eventually death [35]. Therapeutic approaches that aim at ameliorating sepsis survival by dampening systemic inflammation have failed so far [36], demonstrating the necessity to prevent exacerbated inflammation without impairing the host’s immune system.

In the current study we investigated whether immunomodulation during chronic filarial infection impacts protective immune responses against an acute bacterial infection in vivo. Therefore, we used the rodent filarial nematode *Litomosoides sigmodontis* that induces chronic infections in susceptible BALB/c mice, harbors *Wolbachia* bacteria [37], and is a well-established model for human filariasis [38]. Survival, development of hypothermia, bacterial load and systemic levels of pro-inflammatory cytokines were determined to assess *Escherichia coli*-induced sepsis severity, a human relevant pathogen. In this setting, mice deficient in IL-4, IL-4Rα-signaling and TLR2 were used to clarify the impact of AAM and TLR2/*Wolbachia*-induced macrophage modulation, respectively. Our study reveals that chronic *L. sigmodontis* infection has a beneficial effect on an acute bacterial infection by promoting bacterial clearance and macrophage phagocytosis. Interestingly, induction of AAM was dispensable for the protective effect of *L. sigmodontis* infection on *E. coli*-induced peritoneal sepsis, whereas TLR2-triggering during the reprogramming of functional macrophages was essential.

## Results

### Chronic *L. sigmodontis* infection improves *E. coli*-induced sepsis outcome

Chronic filarial infections induce regulatory, anti-inflammatory immune responses in their hosts. In order to investigate whether filariae-induced immunomodulation alleviates acute pro-inflammatory immune responses that develop during sepsis, *L. sigmodontis*-infected BALB/c mice (90 dpi) were intraperitoneally challenged with *E. coli*. Development of *E. coli*-induced hypothermia was monitored over time and peritoneal bacterial load was determined. Six hours after *E. coli* challenge, cytokine and chemokine concentrations were measured from serum and peritoneal cell populations and their activation status were analyzed by flow cytometry.

**Figure 1:**
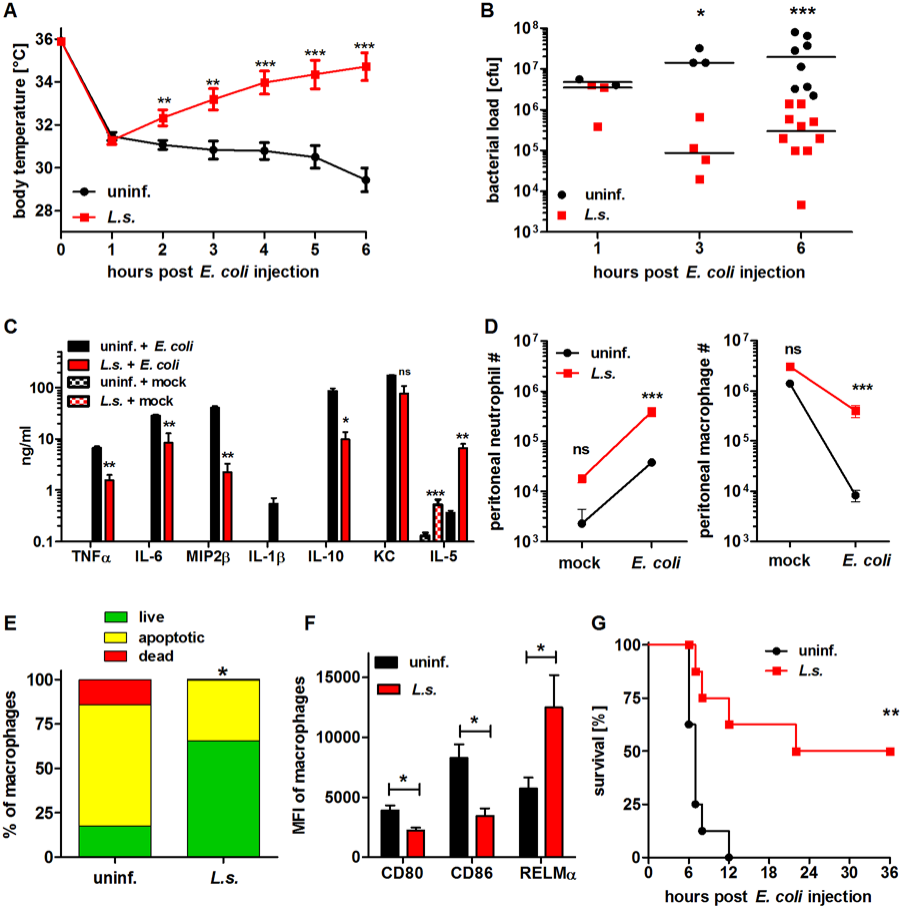
Chronic *L. sigmodontis* infection improves *E. coli*-induced sepsis. (**A**) Kinetic of body temperature in response to i.p. *E. coli* injection of uninfected (n = 17) and chronically *L. sigmodontis* (*L.s.*)-infected mice (n = 19). (**B**) Peritoneal bacterial load and (**C**) serum cytokine/chemokine concentrations (n = 5–6/group) of uninfected and *L. sigmodontis-*infected mice at indicated time points and six hours post *E. coli* injection or mock injection, respectively. (**D**) Total number of peritoneal Gr1^+^ neutrophils and F4/80^hi^CD11b^hi^ macrophages six hours post injection of *E. coli* or sterile LB broth (indicated as mock; n = 4–6/group). (**E**) Frequencies of propidium iodide-positive, Annexin V-positive and double negative (live) F4/80^+^ macrophages three hours post *E. coli* challenge. (**F**) CD80, CD86 and RELMα expression (mean fluorescence intensity) on F4/80^hi^ macrophages (n = 4/group) six hours post *E. coli* injection; (**G**) survival of naïve and *L. sigmodontis*-infected mice after i.p. injection with *E. coli* (n = 8/group). (**A**) Shows pooled data from two independent experiments and (**B-D, F**) show one representative dataset of three independent experiments. Data in (**A**) is displayed as mean +/- SEM and was tested for statistical significance by 2-way ANOVA and Bonferroni post hoc test; data in (**B-F**) was tested for statistical significance by Mann-Whitney-U-test. Indicated statistical significant differences in (**C**) are based on comparisons of *E. coli*-treated groups and, in the case of IL-5, non-*E. coli*-treated groups (*p<0.05, **p<0.01, ***p<0.001); (**G**) was tested for statistical significance by Mantel-Cox Log-rank test (p = 0.0016).

As soon as one hour after *E. coli* challenge, both *L. sigmodontis*-infected and uninfected mice developed hypothermia which continuously decreased in *L. sigmodontis*-uninfected mice over the course of 6 hours post *E. coli* injection. In contrast, chronically infected *L. sigmodontis* mice regained their initial body temperature within the monitored 6h (Fig. 1A). Lessened *E. coli*-induced hypothermia in *L. sigmodontis*-infected mice correlated with a significant reduction in the peritoneal bacterial load after 3h and 6h (6h post *E. coli*: *L. sigmodontis*-infected: spearman r = -0.56, p<0.0001; uninfected: spearman r = -0.68, p<0.0001; Fig. 1B). Serum concentrations of pro-inflammatory cytokines and chemokines (TNFα, IL-6, MIP-2β/CXCL2, IL-1β and KC/CXCL1) as well as IL-10 (p>0.05) were all significantly reduced in the infected group when compared to uninfected *E. coli* controls (Fig. 1C). *E. coli*-injected, *L. sigmodontis*-infected mice did, however, present increased levels of IL-5 in sera when compared to *E. coli*-treated, uninfected controls (Fig. 1C). This outcome was not altered when IL-5 levels were determined over infected mice that were not injected with *E. coli* (Fig. 1C), indicating that effects were not due to a simple overall immune suppression. In the absence of *E. coli* challenge, TNFα, IL-6, MIP-2β, IL-1β, IL-10 and KC were neither detected in serum of chronic *L. sigmodontis*-infected nor uninfected, mock-treated mice (Fig. 1C). On the cellular level, bacterial challenge led in both groups to a significant reduction in peritoneal macrophages, whereas recruitment of neutrophils to the peritoneum increased. When compared to *E. coli* control mice macrophage and neutrophil numbers were significantly increased in *L. sigmodontis*-infected mice six hours after *E. coli* challenge (Fig. 1D). The increased number of peritoneal macrophages in *L. sigmodontis*-infected animals following *E. coli* challenge correlated with reduced frequencies of apoptotic and propidium iodide-positive macrophages in comparison to recovered macrophages from *E. coli*-treated mice (mean 31.2% vs. 82.3%; Fig. 1E). Moreover, 6h after *E. coli* challenge, only 0.6% of the initial number of peritoneal macrophages were left in the *E. coli*-uninfected group, while in *L. sigmodontis*-infected mice, 13.1% of peritoneal macrophages were still present. However, the overall reduced number of macrophages was higher in the *L. sigmodontis*-infected group (on average 1.4×10^6^ vs. 2.6×10^6^ peritoneal macrophages). In order to analyze whether the changes in peritoneal macrophage numbers after *E. coli* challenge in *L. sigmodontis*-infected and uninfected mice correlated with a different phenotype and activation status, expression levels of CD80, CD86 and RELMα were analyzed by flow cytometry (gating scheme of RELMα is shown in S1A Fig.). After *E. coli* challenge, peritoneal macrophages from *L. sigmodontis*-infected mice expressed significantly lower levels of the co-stimulatory molecules CD80 and CD86, but higher levels of RELMα (Fig. 1F), a marker generally associated with AAM. This elevation in RELMα expression was only observed in *L. sigmodontis*-infected animals when they were exposed to *E. coli* (S1B Fig.).

To demonstrate that chronic filarial infection improves sepsis survival, mice were challenged with an increased dose of 6×10^8^ *E. coli* cfu and survival was monitored. Survival experiments revealed that 50% of the *L. sigmodontis*-infected mice survived a severe *E. coli*-induced sepsis, whereas all *E. coli*-only treated controls died within twelve hours (p<0.01; Fig. 1G).

Taken together these results indicate that concurrent *L. sigmodontis* infections substantially improve both survival and infection parameters of *E. coli*-induced sepsis. The *L. sigmodontis*-mediated protective effect was further correlated with increased numbers of phagocytes at the site of bacterial challenge and the fact that these macrophages presented a less activated phenotype.

### *L. sigmodontis*-mediated protective effect is dependent on macrophages

**Figure 2:**
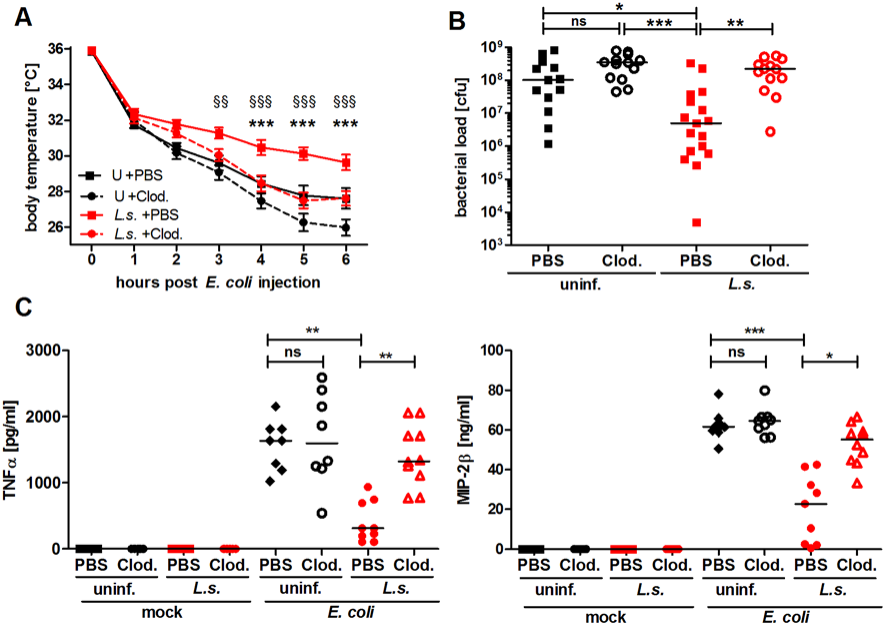
Macrophage depletion renders *L. sigmodontis*-infected mice susceptible to *E. coli*-induced sepsis. Chronic *L. sigmodontis* (*L.s.*)-infected BALB/c mice and uninfected controls (U) were i.p. injected with Clodronate- or PBS-containing liposomes before *E. coli* injection. (**A**) Kinetic of *E. coli*-induced hypothermia. (**B**) Peritoneal bacterial load and (**C**) serum TNFα and MIP-2β levels six hours after *E. coli* injection or mock treatment. (**A**) and (**B**) show pooled data from three independent experiments with at least 4 mice per group. (**C**) Pooled data from two independent experiments (mock treatment single experiment). Data in (**A**) is displayed as mean +/- SEM and was tested for statistical significance by 2-way ANOVA and Bonferroni post-hoc test (asterisks indicate significant differences between *L.s.* + PBS (n = 17) and *L.s.* + Clod. (n = 16), paragraphs indicate significant differences between *L.s.* + PBS and U + PBS (n = 15) treated mice (U + Clod. n = 13); in (**B**) and (**C**) data is presented as median and was tested for statistical significance by 1-way ANOVA followed by Dunn’s post-hoc test comparing only *E. coli*-treated groups. *p<0.05, **p<0.01, ***p<0.001.

To demonstrate that macrophages are essential for the improved sepsis outcome in *L. sigmodontis*-infected mice, we used clodronate containing liposomes to deplete macrophages in vivo. Successful macrophage depletion was confirmed using flow cytometry (S2A Fig.). Macrophage depletion exacerbated *E. coli*-induced hypothermia and no longer reduced the peritoneal bacterial load in *L. sigmodontis*-infected mice compared to PBS-loaded liposome-treated *L. sigmodontis*-infected controls (Fig. 2A, B). In association, serum TNFα and MIP-2β levels were significantly increased in macrophage-depleted, *L. sigmodontis*-infected animals six hours after *E. coli* injection (Fig. 2C). Macrophage depletion in uninfected groups worsened *E. coli*-induced hypothermia 5–6h following *E. coli* challenge (Fig. 2A), but had no statistical impact on bacterial clearance nor levels of TNFα and MIP-2β in sera (Fig. 2B, C). In the absence of *E. coli* challenge, clodronate depletion did not induce the release of detectable concentrations of TNFα and MIP-2β in serum (Fig. 2B, C). Furthermore, although clodronate depletion moderately reduced peritoneal monocyte numbers (S2B Fig.), treatment had no impact on peritoneal neutrophil numbers (S2C Fig.). Collectively, the results demonstrate that *L. sigmodontis* mediates protective effects against an *E. coli*-induced sepsis in a macrophage dependent manner.

### Chronic *L. sigmodontis* infection lessens *E. coli*-induced pro-inflammatory gene expression profiles of peritoneal macrophages

**Figure 3:**
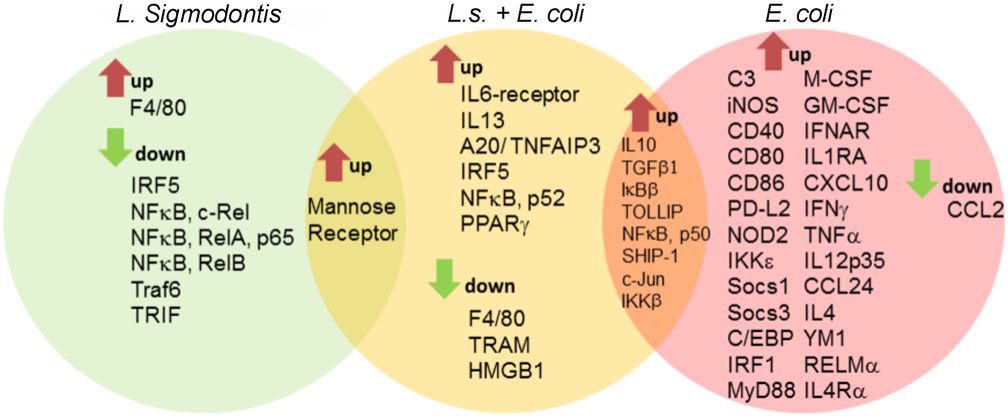
Macrophage transcriptional analysis reveals a less inflammatory phenotype in *L. sigmodontis*-infected mice during *E. coli* challenge. Gene expression profiles of peritoneal macrophages from *L. sigmodontis* (*L.s.*)-infected (left), *L.s.*-infected and *E. coli* challenged (middle) as well as *E. coli* challenged control mice (right) following PCR array analysis. Shared and exclusively regulated genes among the experimental groups were analyzed in comparison to gene expression of naïve control macrophages (cut-off: p<0.05 and >2-fold change). Statistical significances were tested by student’s t-test of the replicate 2^ (-Delta Ct) values for each gene in the control group and treatment groups.

Given that macrophages were essential for the observed protective outcome against *E. coli* challenge in *L. sigmodontis*-infected mice, a customized PCR array was used to identify differences in gene expression profiles of peritoneal macrophages before and three hours after *E. coli* challenge. Fig. 3 depicts genes that were significantly up- and downregulated (min. two fold change) in macrophages from *L. sigmodontis*-infected, *L. sigmodontis*-infected with *E. coli* challenge as well as *E. coli*-only challenged mice compared to macrophages from mock-treated controls. The complete gene list including fold-change and p-values is provided in S1 Table. Differentially expressed genes in Fig. 3 were hereby grouped for exclusive and shared regulation among the three groups. *L. sigmodontis* infection, in the absence of an *E. coli* challenge, led to a significant downregulation of the TLR signaling components Traf6 and TRIF as well as the transcription factors IRF5 and NFκB (RelA, RelB and c-Rel), suggesting the induction of a hypo-responsive macrophage phenotype in terms of TLR activation. Macrophages derived from *E. coli*-only challenged mice exhibited a strong pro-inflammatory gene expression profile as demonstrated by the significant up-regulation of genes such as iNOS, CD40, CD80, CD86 and pro-inflammatory cytokines/chemokines (TNFα, IL-12p35, IFNγ and CXCL10). Simultaneously, signaling components known for their anti-inflammatory properties were induced (PD-L2, Socs1, Socs3, IL-4, YM1, RELMα, IL-4Rα). Consistent with our ex vivo findings, macrophages from co-infected mice did not show a significant up-regulation of genes associated with classically activated macrophages on the mRNA level. Shared macrophage genes up-regulated in both *E. coli*-only and co-infected animals were associated with anti-inflammatory and regulatory functions in the TLR signaling pathway (IL-10, TGFβ, IκBβ, TOLLIP, SHIP). Genes exclusively up-regulated in co-infected mice were three transcription factors (IRF5, NFκB-p52 and PPARγ), A20/TNFAIP3, the IL-6-receptor and IL-13. Exclusively downregulated genes in the co-infected group were F4/80 (adhesion), TRAM (TLR signaling) and HMGB1 (inflammatory mediator, TLR4-binding). Only one gene in the panel (mannose receptor) was significantly up-regulated in both *L. sigmodontis*-mono and co-infected mice. Importantly, no genes were co-regulated in macrophages of *L. sigmodontis*-only and *E. coli*-only infected mice, demonstrating the extent to which the respective responses to each infection differed. A direct comparison of macrophage gene expression profiles in co-infected and *E. coli*-only treated mice surprisingly revealed a reduced expression of some markers of AAM activation (CD273/PD-L2 (p<0.05), RELMα (p>0.05), but not YM1) in macrophages from co-infected mice. In addition, there was a significant reduction in TGFβ expression as well as the type 2 cytokines IL-4, but an increased expression of NFκB RelB and TLR1 in macrophages from co-infected mice (S2 Table). Macrophages isolated from the co-infected group preferentially expressed genes of bactericidal effectors (iNOS, C3) and regulators of TLR/NFκB signaling (IRAK-M, IKKε, SOCS3, PPARȏ), whereas markers of pro-inflammatory responses (CD40, CD80, CD86, IFNγ, CXCL10, IRF1) were predominantly induced in *E. coli*-only challenged mice, albeit not statistically significant.

### Filariae-induced AAM are not required for improved sepsis outcome

*L. sigmodontis* infection led to increased numbers of peritoneal RELMα-positive macrophages after *E. coli* challenge as determined by flow cytometry, but did not induce a gene expression profile that corresponded with typical AAM. To clarify whether AAM were involved in the *L. sigmodontis*-mediated protection against an *E. coli*-induced sepsis, we used IL-4Rα/IL-5 double deficient BALB/c mice—which do not develop AAM [39], and IL-4-deficient BALB/c mice, which lack the suppressive effect of AAM [40].

**Figure 4:**
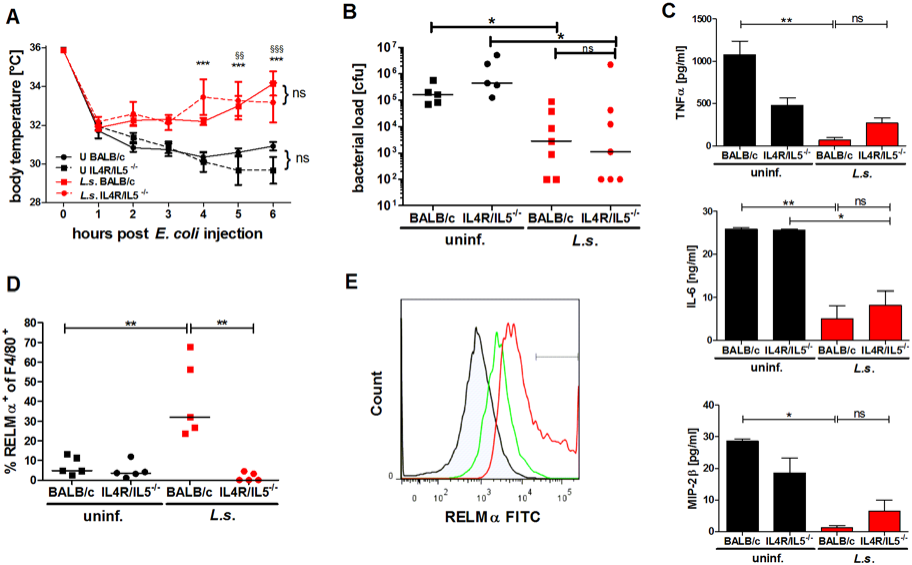
*L. sigmodontis*-mediated protection against *E. coli*-induced sepsis is not compromised in AAM-deficient IL-4Rα^-/-^/IL-5^-/-^ mice. **(A)** Kinetic of body temperature in response to i.p. *E. coli* injection of uninfected (U) and chronic *L. sigmodontis* (*L.s.*)-infected wild type and IL-4Rα/IL-5-deficient mice. **(B)** Peritoneal bacterial load, **(C)** serum concentrations of TNFα, IL-6 and MIP-2β and **(D)** frequency of F4/80-positive peritoneal macrophages that are RELMα positive in those animals six hours post *E. coli* injection. **(E)** Representative histogram of RELMα fluorescence intensity of F4/80-positive peritoneal macrophages (isotype control (shaded), *L.s.*-infected IL-4Rα/IL-5-deficient mice (green), *L.s.*-infected wild type mice (red)). (**A-D**) Representative dataset from one of two independent experiments with at least 5 mice per group. Data shown in (**A**) is displayed as mean +/- SEM and was tested for statistical significance by 2-way ANOVA and Bonferroni post-hoc test (asterisks indicate significant differences between *L.s.*-infected and uninfected IL-4R/IL-5ko mice and paragraphs between *L.s.*-infected and uninfected wild type mice). (**B, C**) and (**D**) data was tested for statistical significance by 1-way ANOVA followed by Dunn’s post-hoc test. *p<0.05, **p<0.01.

Both, *L. sigmodontis*-infected wild type and IL-4Rα/IL-5^-/-^ mice had an ameliorated hypothermia following *E. coli* application when compared to control groups and moreover, displayed a significantly reduced peritoneal bacterial load (Fig. 4A, B). When compared to the corresponding non-infected *E. coli* challenged controls, both *L. sigmodontis*-infected wild type and IL-4Rα/IL-5-deficient mice had reduced systemic TNFα, IL-6 and MIP-2β concentrations (Fig. 4C). As expected, RELMα-positive macrophages were absent in the peritoneum of *L. sigmodontis*-infected IL-4Rα/IL-5-deficient mice (Fig. 4D, E). However, the recruitment of neutrophils in either co-infected group remained unaltered (S3 Fig.). Similarly to the improved outcome in wild type and IL-4Rα/IL-5-deficient *L. sigmodontis*-infected mice, IL-4 deficiency did not impair *L. sigmodontis*-mediated protective effects against *E. coli* challenge since infected IL-4^-/-^mice presented both improved hypothermia and reduced peritoneal bacterial load (S4A-S4B Fig.). These data demonstrate that the induction of RELMα-positive macrophages during *L. sigmodontis* infection is dispensable for an improved outcome of *E. coli*-induced sepsis.

### Endosymbiotic *Wolbachia* modulate macrophage function in a TLR2 dependent manner

Similar to most human pathogenic filariae, *L. sigmodontis* harbors endosymbiotic *Wolbachia* that can be sensed via TLR2, but not TLR4 [41]. The TLR2 dependency of *Wolbachia*-mediated macrophage modulation was investigated in vitro using a *Wolbachia*-containing crude extract from *L. sigmodontis* adult worms (LsAg) and a preparation of the C6/36 insect cell line infected with *Wolbachia*.

Thioglycollate-induced macrophages derived from wild type, TLR2- and TLR4-deficient BALB/c mice were cultured and their activation was followed after stimulation with either *E. coli-*derived LPS (TLR4 ligand), Pam_3_Cys (TLR1/2 ligand), FSL1 (TLR2/6 ligand), LsAg, Ls-tet (an extract derived from *Wolbachia*-depleted *L. sigmodontis* worms) and preparations from sterile (C6/36) and *Wolbachia*-infected (Wolb) insect cell lines.

**Figure 5:**
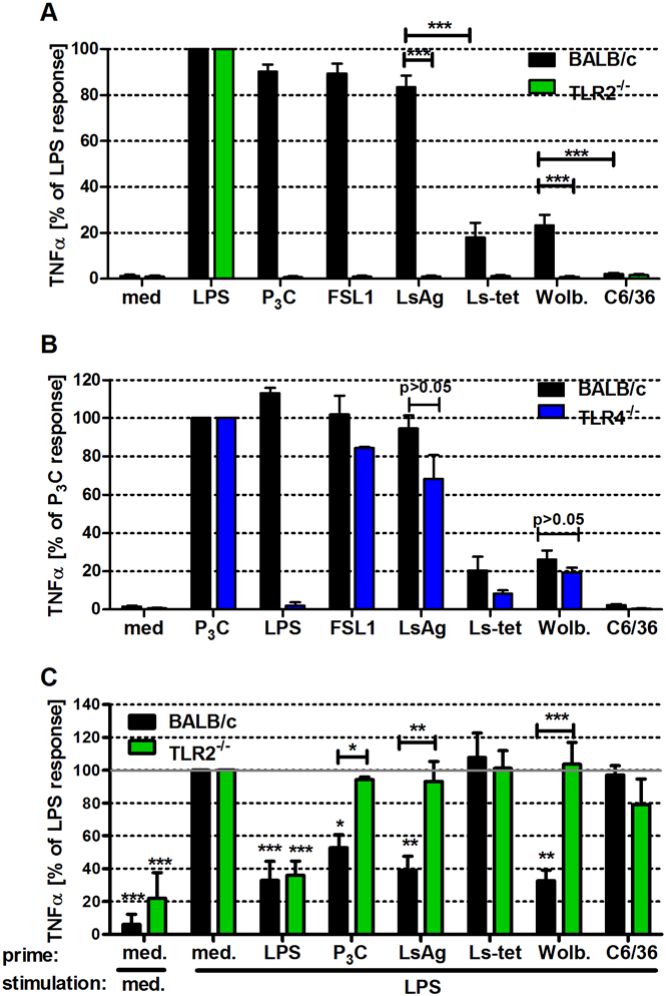
Macrophage stimulation with *Wolbachia*-containing preparations induces TLR2-dependent TNFα secretion and tolerance to subsequent LPS stimulation. Thioglycollate-elicited peritoneal macrophages from wild type and either TLR2- (**A**), or TLR4-deficient (**B**) mice were stimulated with TLR4- (*E. coli*-LPS) and TLR2- (P_3_C, FSL1) specific ligands, *L. sigmodontis* adult worm extract (LsAg), LsAg from *Wolbachia*-depleted worms (Ls-tet), *Wolbachia*-infected (Wolb.) and sterile preparations of C6/36 insect cells. TNFα concentrations relative to LPS (**A**) and P_3_C (**B**) responses are shown. (**C**) Thioglycollate-induced macrophages from wild type and TLR2-deficient mice were primarily stimulated (prime) for 18 hours as described above before re-stimulation with LPS for an additional 18 hours. TNFα concentrations in supernatants of F4/80^hi^ macrophages are plotted relative to the acute LPS response (medium prime/LPS stimulation). Shown are data representative of three independent experiments (n = 5/group for (**A, B**) and n = 3/group for (**C**)). Data shown is expressed as mean + SEM and was tested for statistical significance by student’s t-test. Asterisks above the bars indicate significant differences compared to med/LPS condition. *p< 0.05; **p< 0.01; ***p<0.001.

*Wolbachia*-containing LsAg and Wolb induced TNFα (Fig. 5A) secretion from macrophages in a TLR2-dependent manner, whereas *Wolbachia*-free preparations (Ls-tet and C6/36) induced this cytokine at significantly lower levels (Fig. 5A). *Wolbachia*-triggered cytokine production was comparable in macrophages derived from TLR4-deficient and wild type mice (Fig. 5B). Thus, LsAg and accordingly *L. sigmodontis* adult worms contain *Wolbachia*-derived TLR2 ligands that trigger pro-inflammatory cytokine production from macrophages in a TLR2-, but not TLR4-dependent manner.

Next, we investigated whether exposure of macrophages to *Wolbachia*-derived TLR2 ligands modulated subsequent immune responses. In short, macrophages were pre-incubated for 18h with *Wolbachia*-containing preparations (LsAg, Wolb) and then challenged with LPS. Prior exposure to *Wolbachia*-containing extracts elicited significantly lower amounts of TNFα (Fig. 5C) and expression levels of MHC-II and CD40 (S5A–S5B Fig.). Interestingly, macrophages derived from TLR2-deficient mice did not display this modulated phenotype (Fig. 5C and S5A–S5B Fig.). The results suggest that pre-exposure to TLR2 ligands from *Wolbachia* elicits a cross tolerance that impairs subsequent stimulation by TLR4 ligands.

### *L. sigmodontis* infection has no beneficial effect on sepsis in TLR2-deficient hosts

Given that *Wolbachia* modulated macrophage function in vitro and pre-exposure to *Wolbachia* reduced macrophage activation in response to LPS in a TLR2 dependent manner, we tested whether the *L. sigmodontis*-mediated protective effect against an *E. coli* challenge was dependent on TLR2 signaling.

**Figure 6:**
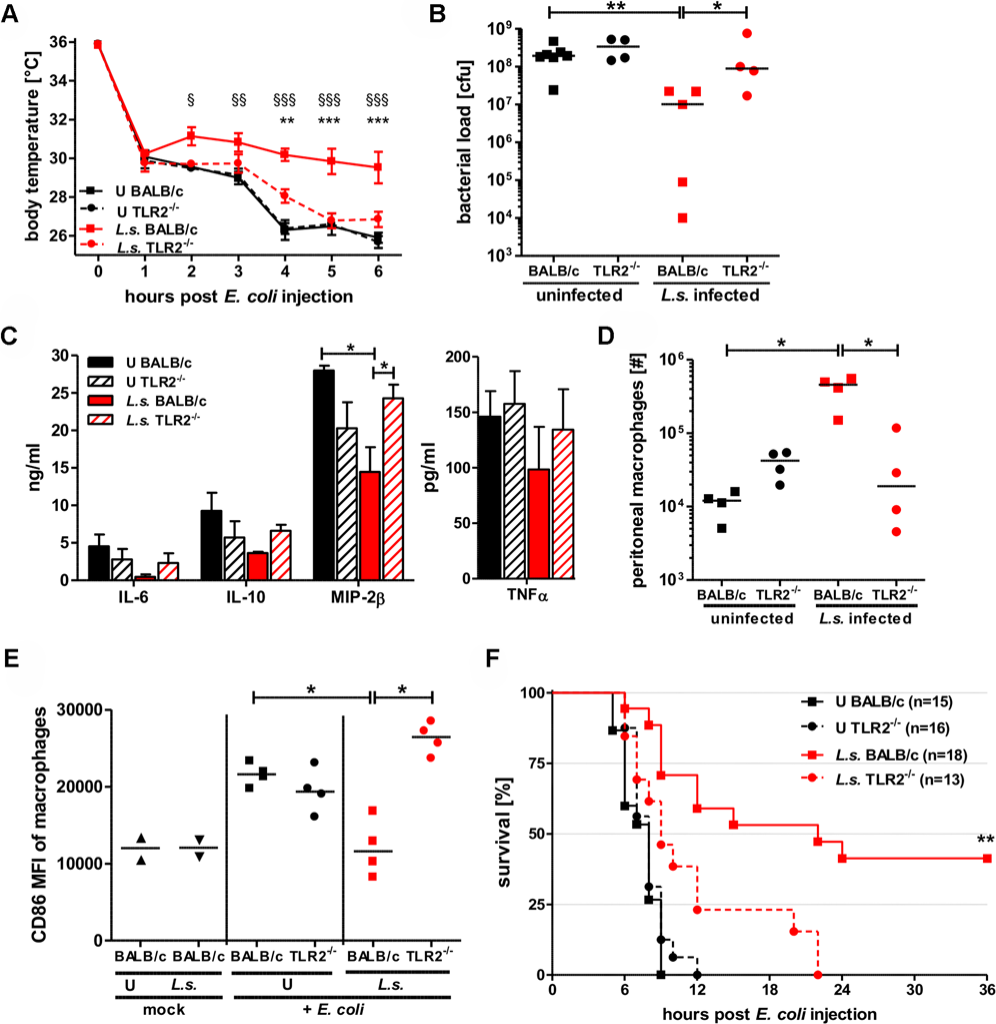
TLR2 is required for mediating protection against *E. coli*-induced sepsis in filarial-infected mice. (**A**) Kinetic of body temperature in response to i.p. *E. coli* injection of uninfected (U) and chronic *L. sigmodontis* (*L.s.*)-infected wild type and TLR2-deficient mice. (**B**) Peritoneal bacterial load, (**C**) serum concentrations of IL-6, IL-10, and MIP-2β, and TNFα, (**D**) total peritoneal F4/80^hi^ macrophage numbers and (**E**) their mean fluorescence intensity (MFI) of CD86 six hours post *E. coli* injection. (**F**) Survival after i.p. injection of *E. coli* into uninfected and *L.s.*-infected wild type (U: n = 15; *L.s.*: n = 18) and TLR2-deficient mice (U: n = 16; *L.s.*: n = 13). (**A-D**) shows a representative dataset from one of two independent experiments with at least 4 mice per group. Data shown in (**A**) is displayed as mean +/- SEM and was tested for statistical significance by 2-way ANOVA and Bonferroni post-hoc test (paragraphs indicate significant differences between *L.s.*-infected and uninfected wild type mice and asterisks in comparison to *L.s.*-infected TLR2^-/-^ mice). Data shown in (**B-E**) was tested for statistical significance by 1-way ANOVA followed by Dunn’s post-hoc test. (**F**) Pooled data from two independent experiments. Differences between *L.s.*-infected BALB/c and TLR2^-/-^ mice were tested for statistical significance by Mantel-Cox Log-rank test (p = 0.0035). *p<0.05; **p<0.01; ***p<0.001.

Analysis of parasite burdens in *L. sigmodontis*-infected wild type BALB/c and TLR2-deficient mice were comparable in terms of adult worm numbers (1–5 adult worms per mouse) and microfilaremia 90 dpi (TLR2^-/-^: 4/10 mice microfilaremic (0–26 microfilariae/50µl blood; wild type: 4/11 mice microfilaremic (0–27 microfilariae/50µl blood). However, the *L. sigmodontis*-mediated protective effect against an *E. coli*-induced sepsis was lost in infected TLR2^-/-^ mice. Six hours after *E. coli* injection, body temperature was significantly lower in *L. sigmodontis*-infected TLR2^-/-^ mice when compared to infected wild type controls (Fig. 6A). In association, *L. sigmodontis* infection in TLR2^-/-^ mice resulted in bacterial loads (Fig. 6B) and levels of pro-inflammatory mediators (Fig. 6C) that were comparable to non-infected *E. coli*-exposed wild type controls. The increased peritoneal macrophage numbers, observed six hours post *E. coli* injection in *L. sigmodontis*-infected wild type mice was not detected in infected TLR2^-/-^ mice (Fig. 6D). Macrophages derived from *L. sigmodontis*-infected wild type mice showed expression levels of CD86 similar to those derived from mice that did not receive a bacterial challenge. Following *E. coli* challenge, CD86 macrophage expression from wild type and TLR2^-/-^ *E. coli*-exposed mice were dramatically increased. While *E. coli*-induced CD86 expression was reduced in *L. sigmodontis*-infected wild type mice, it remained high in infected TLR2^-/-^ mice (Fig. 6E).

Consistent with the results on hypothermia, bacterial clearance and macrophage responses, *L. sigmodontis* infection did not improve sepsis survival in the TLR2-deficient group. All *L. sigmodontis*-infected TLR2-deficient mice died within 22 hours post *E. coli* challenge, whereas 8 out of 18 *L. sigmodontis*-infected wild type mice survived (p<0.01; Fig. 6F). All of the non-infected *E. coli*-challenged wild type and TLR2^-/-^ mice died within twelve hours of bacteria injection (Fig. 6F).

Taken together, these experiments demonstrate that the *L. sigmodontis*-mediated protective effects against an *E. coli*-induced sepsis are dependent on TLR2 signaling. The following sections therefore investigated whether such protection was mediated through *Wolbachia* triggering of TLR2.

### Ameliorated anti-bacterial activity during *L. sigmodontis* infection is dependent upon TLR2 signaling

Bacterial clearance in *L. sigmodontis*-infected mice was significantly improved when compared to uninfected controls and furthermore depended on macrophages and TLR2 expression. To gain insight into how the bacterial load could be reduced in *L. sigmodontis*-infected mice, we compared the phagocytic capacity of macrophages isolated from *L. sigmodontis*-infected and uninfected wild type and TLR2-deficient mice.

In brief, mice were challenged i.p. with *E. coli* for three hours. Equal numbers of isolated macrophages were then cultured in vitro in the presence of gentamycin for an additional four hours. As gentamycin does not penetrate the cell wall, only extracellular bacteria are exposed and eliminated. Thus, by lysing the macrophages and growing the resulting lysate on suitable culture plates, the number of in vivo phagocytosed bacteria can be quantified.

**Figure 7:**
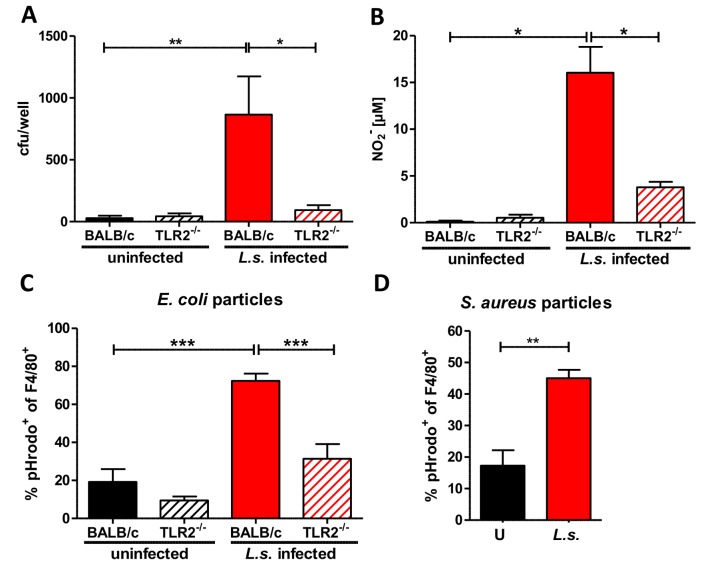
Anti-bacterial effector mechanisms are enhanced by *L. sigmodontis* infection in a TLR2 dependent manner. (**A**) Colony forming units (cfu) obtained by a gentamycin assay using peritoneal macrophages derived from chronic *L. sigmodontis* (*L.s.*)-infected wild type and TLR2^-/-^ mice and respective uninfected controls three hours after i.p. *E. coli* injection. (**B**) Nitrite concentrations of the same macrophages as in (**A**) after ex vivo cultivation for 48 hours. Frequency of peritoneal F4/80^-^positive macrophages from *L.s.-*infected and uninfected wild type and TLR2^-/-^ mice (n = 5 per group) that phagocytosed pHrodo *E. coli*-BioParticles 90 minutes post injection (**C**) or from *L.s.*-infected and uninfected wild type mice (n = 5 per group) that phagocytosed pHrodo *S. aureus*-BioParticles three hours post injection (**D**). (**A**) Pooled data from two independent experiments with at least four mice per group. Data shown in (**A-C**) is illustrated as mean + SEM and was tested for statistical significance by 1-way ANOVA followed by Dunn’s multiple comparisons test; Data in (**D**) is also shown as mean + SEM and was tested for statistical significance by Mann-Whitney U test. *p< 0.05; **p< 0.01; ***p<0.001.

The gentamycin assay revealed that the bacterial content in macrophages from *E. coli*-only challenged mice, regardless of background origin, were equally low, whereas macrophages from *L. sigmodontis*-infected wild type mice harbored significantly more *E. coli* (Fig. 7A). This increased uptake of bacteria was not observed in macrophages recovered from *L. sigmodontis*-infected TLR2^-/-^ mice (Fig. 7A). In vitro generated nitrite in response to *E. coli* was also significantly increased in macrophages derived from *L. sigmodontis*-infected wild type mice compared to macrophages from uninfected and *L. sigmodontis*-infected TLR2^-/-^ controls (Fig. 7B).

To confirm the improved bacterial uptake by macrophages from *L. sigmodontis*-infected animals, mice were challenged i.p. with pHrodo *E. coli* particles. These particles are pH sensitive and fluoresce in an acidic environment like the phagolysosome. This fluorescence intensity allows one to distinguish particles that are bound to the cell surface from those that are engulfed by phagocytes and fused with the phagolysosome. Flow cytometric analysis revealed that *L. sigmodontis*-infected mice had a significantly higher frequency of fluorescent pHrodo-positive F4/80^+^ macrophages 90 min (Fig. 7C) after *E. coli* particle challenge compared to macrophages derived from uninfected control groups. The improved uptake of *E. coli* particles was not observed by macrophages isolated from *L. sigmodontis*-infected TLR2-deficient mice (Fig. 7C). In similar assays, macrophages from *L. sigmodontis*-infected wild type mice had an enhanced uptake of fluorescently labeled *Staphylococcus aureus* particles 3h (Fig. 7D) after injection, suggesting that the improved bacterial uptake is not restricted to gram-negative bacteria or TLR4 signaling. Thus, in a TLR2-dependent manner, macrophages from *L. sigmodontis*-infected animals are more efficient in the uptake of bacteria and produce higher amounts of bactericidal nitric oxide.

### Repeated administration of *Wolbachia*-containing preparations and TLR2 ligands improves *E. coli*-induced sepsis

**Figure 8:**
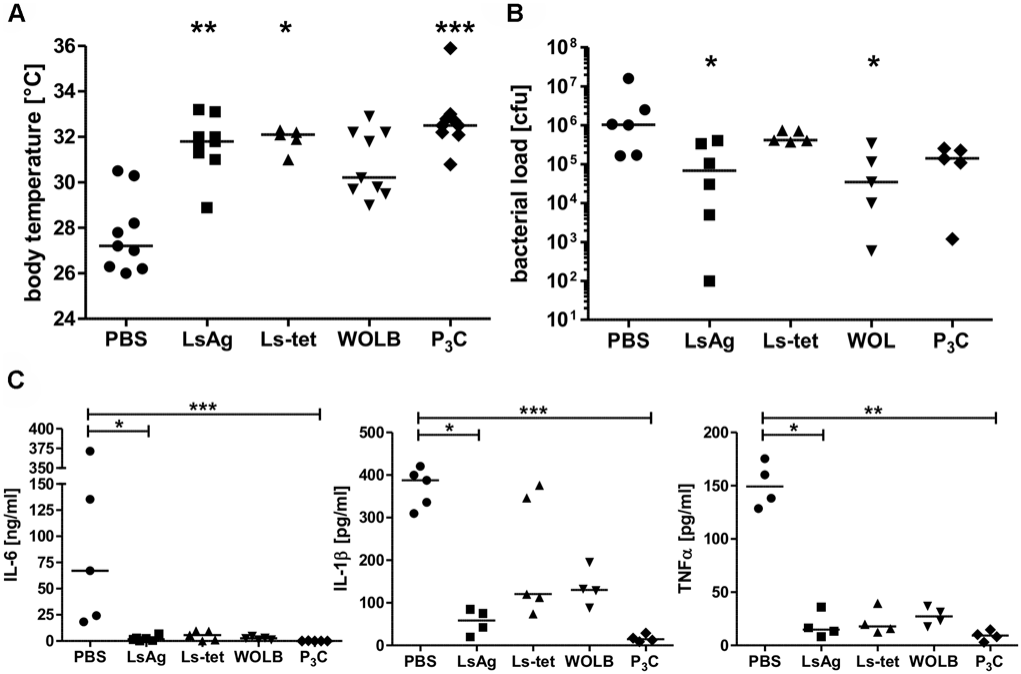
Repeated injections of *Wolbachia*-containing preparations and TLR2 ligands improve subsequent *E. coli*-induced sepsis *in vivo*. Mice were injected three times with 40µg of *L. sigmodontis* extract (LsAg), *L. sigmodontis* extract obtained from tetracycline treated worms (Ls-tet), *Wolbachia* (WOLB) or Pam_3_Cys (P_3_C) every fourth day. Two days after the last injection sepsis was induced by i.p. *E. coli* injection. Body temperature (**A**), bacterial loads (**B**) and serum IL-6, IL-1β, and TNFα levels (**C**) were monitored six hours after *E. coli* challenge. Data in (**A-C**) is depicted as median and was tested for statistical significances using Kruskal-Wallis followed by Dunn’s multiple comparisons test.*p< 0.05;.**p< 0.01; ***p<0.001.

Next, we tested whether administration of *Wolbachia*-containing reagents prior to sepsis could improve disease outcome. Thus, naïve mice were injected three times on four day intervals with either LsAg, LsAg without *Wolbachia* (Ls-tet), *Wolbachia*, Pam_3_Cys or PBS alone. Two days after the last injection, peritoneal sepsis was induced. Serial injections of LsAg, *Wolbachia* and Pam_3_Cys improved *E. coli*-induced hypothermia (Fig. 8A), bacterial clearance (Fig. 8B) and reduced serum IL-6, IL-1β, and TNFα concentrations (Fig. 8C) compared to PBS controls. Although the administration of Ls-tet had a beneficial effect on *E. coli*-induced hypothermia (Fig. 8A), it had little effect on bacterial load (Fig. 8B). The administration of this extract did however reduce serum levels of IL-6, IL-1β, and TNFα (p>0.05; Fig. 8C). These experiments demonstrate that priming mice with LsAg per se or *Wolbachia* and synthetic TLR2 ligands protects them from *E. coli*-induced sepsis. Moreover, there appears to be additional *Wolbachia*-independent protective mechanisms since the application of Ls-tet antigen also benefited the mice to some degree.

### Transfer of primed macrophages improves sepsis outcome

To directly test the beneficial effect of *Wolbachia*/TLR2-mediated modulation of macrophage function in bacterial sepsis, macrophages from naïve wild type and TLR2^-/-^ mice were pre-treated with LsAg or *Wolbachia* preparations in vitro and then adoptively transferred into naïve mice that subsequently received an i.p. *E. coli* injection.

**Figure 9:**
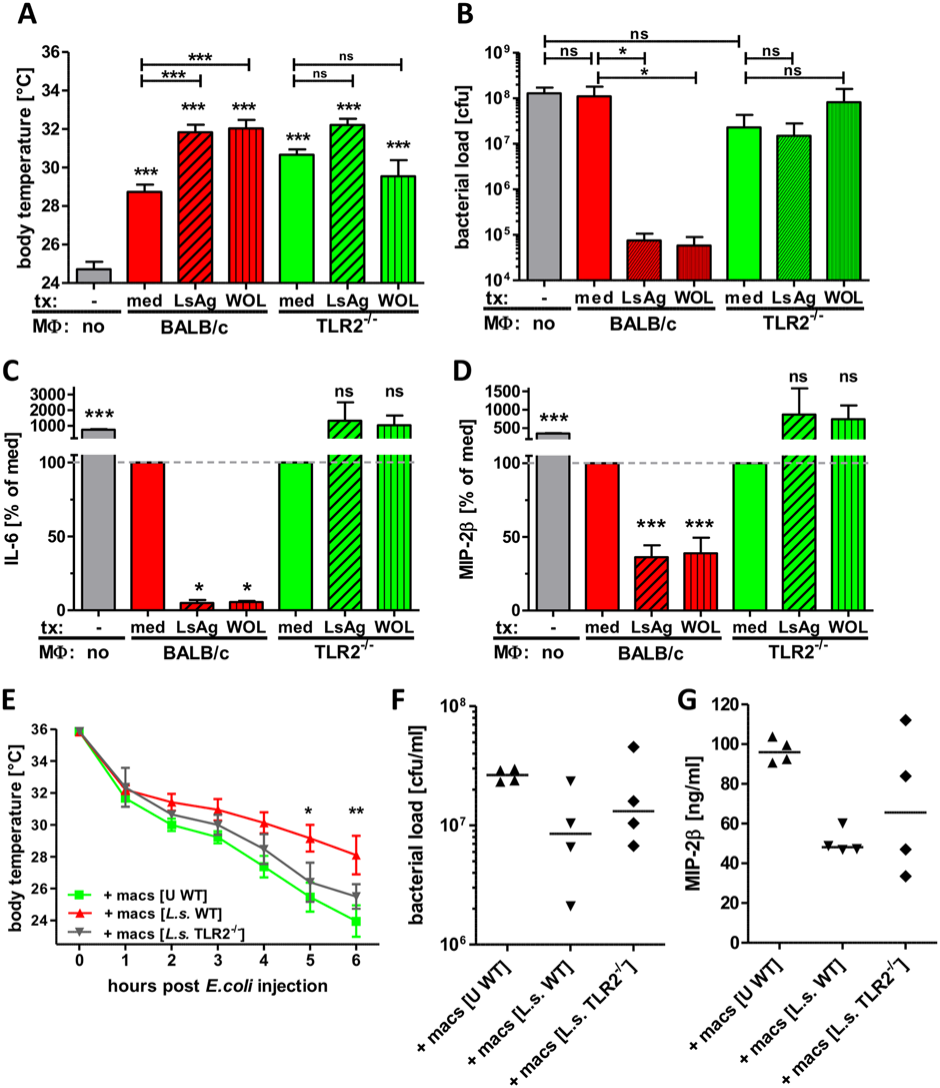
Transfer of primed macrophages improves *E. coli*-induced sepsis. Macrophages derived from Thioglycollate-treated BALB/c or TLR2^-/-^ mice were pre-treated *in vitro* with LsAg, *Wolbachia* or medium for 24 hours and subsequently transferred into naïve recipient wild type mice (n = 6/group). 12 hours after transfer sepsis was induced by i.p. *E. coli* injection. Body temperature (**A**), peritoneal bacterial loads (**B**) and serum IL-6 (**C**) and MIP-2β (**D**) were measured six hours after *E. coli* challenge. In a separate experiment wild type (WT) mice received peritoneal macrophages from either uninfected (U) wild type or chronic *L. sigmodontis* (*L.s.*)-infected WT or TLR2-deficient mice 12h before sepsis. Kinetic of body temperature (**E**), peritoneal bacterial loads (**F**) and serum MIP-2β (**G**) were measured six hours after *E. coli* challenge. Bar graphs in (**A-D**) show mean + SEM from recipient mice. Statistical significances were determined by 1-way ANOVA followed by Dunn’s multiple comparisons test. Data in (**E**) is displayed as mean +/- SEM and was tested for statistical significance by 2-way ANOVA and Bonferroni post-hoc test. Symbols in (**F, G**) show individual values of recipient mice that were tested for statistical significances by 1-way ANOVA followed by Dunn’s multiple comparisons test. *p< 0.05; **p< 0.01; ***p<0.001.

The transfer of macrophages, regardless of genotype or in vitro pre-treatment improved *E. coli*-induced sepsis of recipient mice compared to PBS-treated controls (Fig. 9), suggesting that enhanced numbers of macrophages at the site of infection have a beneficial effect on sepsis. Nevertheless, a transfer of macrophages pre-treated with LsAg or *Wolbachia* from wild type mice further improved sepsis parameters resulting in significantly milder hypothermia, reduced bacterial burden and lower serum levels of IL-6 and MIP-2β compared to control mice, which received medium pre-treated macrophages (Fig. 9A-D). A transfer of macrophages derived from TLR2-deficient mice resulted in a similar improvement of *E. coli*-induced hypothermia and was independent of the pre-transfer treatment (Fig. 9A). Most strikingly, the transfer of macrophages derived from TLR2-deficient mice had no beneficial effect on the bacterial burden and again, this was independent of their pre-activation stimulus (Fig. 9B). Furthermore, the transfer of macrophages pre-treated with *Wolbachia* or LsAg from TLR2-deficient mice did not reduce serum IL-6 and MIP-2β levels compared to mice that received medium pre-treated macrophages lacking TLR2 (Fig. 9C, D).

To finally demonstrate that macrophages from *L. sigmodontis*-infected animals have a beneficial effect on *E. coli*-induced sepsis, peritoneal macrophages were isolated from chronic *L. sigmodontis*-infected wild type or TLR2-deficient mice as well as naïve controls and transferred into naïve wild type recipients. *E. coli*-induced hypothermia was significantly improved in mice that received macrophages from *L. sigmodontis*-infected wild type mice in comparison to mice that received macrophages from either *L. sigmodontis*-infected TLR2-deficient or uninfected wild type controls (Fig. 9E). Accordingly, the peritoneal bacterial burden and serum MIP-2β levels were lowest in recipients of macrophages derived from *L. sigmodontis*-infected wild type controls (Fig. 9F, G), although those differences did not reach statistical significance. These results provide direct evidence that exposure to *Wolbachia*-containing preparations or *L. sigmodontis* infection induces a unique class of macrophages via TLR2 which can then facilitate bacterial clearance and dampen excessive inflammatory responses during bacterial sepsis.

## Discussion

Macrophages play a pivotal role during sepsis [42, 43] since they not only recognize pathogens via pattern recognition receptors and initiate inflammatory cascades through the release of pro-inflammatory mediators but can eliminate pathogens via phagocytosis and secrete bactericidal reactive oxygen species as well. In the current study, we demonstrate that modulation of macrophage function by either chronic infection with the filarial nematode *L. sigmodontis* or priming with filarial antigens and TLR2 ligands improves *E. coli*-induced sepsis outcome.

Macrophage depletion and transfer experiments in our study clearly demonstrated the significant role of macrophages in sepsis per se. Macrophage depletion worsened *E. coli*-induced hypothermia supporting a protective role of peritoneal macrophages in sepsis, while bacterial clearance and systemic pro-inflammatory cytokine/chemokine levels were not affected. Transfer of thioglycollate-induced unprimed macrophages lessened on the other hand *E. coli*-induced hypothermia and reduced systemic pro-inflammatory cytokine/chemokines levels, but did also not affect bacterial burden. Those findings suggest that macrophages are essentially involved in the protective immune responses against sepsis, but modulation of their function can further promote their protective role.

In recent years it has become apparent that macrophages represent a diverse and plastic entity, capable of initiating and modulating both innate and adaptive immune responses [44, 45]. Macrophage populations are not restricted to either classically activated macrophages or AAM, but can be defined in a continuum of activation types [46, 47]. Our in vitro studies performed here demonstrate that stimulation of peritoneal macrophages with *L. sigmodontis* adult worm extract or *Wolbachia*-containing insect cells induced macrophage activation and pro-inflammatory cytokine release and diminish subsequent LPS-driven macrophage activation in a TLR2- and *Wolbachia*-dependent manner. These findings are in accordance to the study by Turner et al. (2006) which used *Wolbachia*-containing *Brugia malayi* extract [48].

Consistent with macrophage modulation in vitro, gene expression profiling of macrophages from chronic *L. sigmodontis*-infected *E. coli*-challenged mice revealed an up-regulation of the anti-inflammatory cytokines IL-10 and TGFβ as well as negative regulators of TLR signaling and NFκB activation (A20/TNFAIP3, TOLLIP, SHIP-1, IκB), which are all associated with TLR hypo-responsiveness to LPS during endotoxin tolerance [49]. Macrophages from co-infected mice further downregulated the expression of genes involved in TLR ligand responses (HMGB1, F4/80 and TRAM), suggesting a tolerant macrophage phenotype. Whereas genes associated with anti-inflammatory immune responses were regulated in macrophages from *E. coli*-only challenged mice as well (IL-10, TGFβ, SHIP-1, TOLLIP, IκB), the induction of pro-inflammatory cytokines and chemokines (TNFα, IL-12p35, CXCL10, IFNγ) were exclusively regulated in macrophages from *E. coli*-only treated mice. Accordingly, expression levels of the macrophage co-stimulatory molecules CD80 and CD86 by either flow cytometry or PCR array were strongly regulated in macrophages from *E. coli*-only challenged mice and correlated with the pronounced systemic inflammation and the poor sepsis outcome in those animals. In the absence of *E. coli* challenge, macrophages in the peritoneum of chronically infected *L. sigmodontis* mice were only slightly modulated, displaying an increased expression of the mannose receptor gene and downregulation of genes associated with TLR and NFκB activation, while genes that correlate with an AAM phenotype were not increasingly expressed. Increased expression of the mannose receptor, which was previously associated with an AAM phenotype [50] and helminth immunomodulation [51], may hereby account for the improved binding and phagocytosis of pathogens [52] in our study, while the reduced expression of genes downstream of TLR signaling may reduce TLR-induced macrophage activation. Interestingly, six hours post *E. coli* challenge peritoneal macrophages from *L. sigmodontis*-infected mice upregulated RELMα expression, a marker that is expressed on AAM, which may suggest that *L. sigmodontis* infection primes towards an underlying Th2 immune response within the peritoneum that is triggered by *E. coli* challenge. In association, *L. sigmodontis*-infected animals had increased levels of IL-5 in the sera in the absence of an *E. coli* challenge which were further increased following *E. coli* challenge. However, markers associated with AAM were not significantly upregulated in the PCR array three hours after the *E. coli* injection. The complex modulation of macrophage function and gene expression in our study is therefore not in accordance with a typical AAM phenotype and may explain the beneficial effect we observed compared to studies that investigated AAM. While AAMs are generally linked to a higher susceptibility to bacterial infections [20, 53] and inhibit classical macrophage inflammation via arginase 1 production [2], they may have beneficial effects by lessening sepsis induced tissue damage. Reprogramming of AAMs by TLR stimulation can also enhance their microbial killing capacity [54]. Our experiments using mice that are either deficient for the IL-4 receptor alpha, which is required for AAM induction, or deficient for IL-4, which is necessary for the suppressive function of AAM [39, 40], suggest, however, that modulation of NFκB and TLR signaling, but not the induction of an AAM phenotype, improves sepsis outcome in *L. sigmodontis*-infected mice.

Additional characteristics of *L. sigmodontis*-exposed peritoneal macrophages during *E. coli* challenge included the increased release of nitrogen species, as well as an improved phagocytosis promoting bacterial clearance that was not restricted to TLR4 associated pathogens. All these factors correlate with previously described endotoxin tolerant macrophage populations [55, 56]. Reduced *E. coli*-induced activation in macrophages from *L. sigmodontis*-infected mice probably resulted in the reduced frequency of apoptotic and dead peritoneal macrophages compared to *E. coli*-only challenged mice. Findings from the macrophage transfer experiments using equal numbers of in vitro modulated peritoneal macrophages further suggest that tolerogenic responses are more likely to result from intrinsic changes in the macrophages rather than an increased number of apoptotic macrophages.

Next we demonstrated in our study the relevance of TLR2 signaling for the *L. sigmodontis*-mediated protective effect through the fact that mice deficient in TLR2 did not show an improved sepsis outcome when infected with *L. sigmodontis*. This lack of protection was not due to an increased susceptibility of TLR2-deficient mice to *E. coli*, as wild type and TLR2-deficient mice responded equally to an *E. coli* challenge, confirming previous reports [57]. Parasite burden was also unaltered between TLR2-deficient and wild type BALB/c mice, excluding the possibility that differences in *L. sigmodontis* baseline levels were the cause for the reverted sepsis phenotype. We actually revealed that macrophages from *L. sigmodontis*-infected TLR2-deficient mice did not have an improved phagocytic capacity compared to wild type macrophages. In addition, TLR2 signaling was required for in vitro macrophage priming with *Wolbachia* or LsAg. Accordingly, administration of TLR2 ligands, *Wolbachia* and *Wolbachia*-containing LsAg improved bacterial sepsis in vivo. However, the observed partial improvement of *E. coli*-induced sepsis by administration of Ls-tet antigen suggests that protection maybe mediated by residual *Wolbachia*-derived ligands. Alternatively, the observed reduction in the different sepsis parameters could be mediated by distinctive *L. sigmodontis*-derived components of which at least the bacterial load was dependent on TLR2 signaling. In accordance, transfer of macrophages that were not modulated in vitro beforehand resulted in a milder *E. coli*-induced hypothermia and reduced pro-inflammatory cytokine/chemokines levels, but did not impact the bacterial burden. Finally, transfer experiments using in vitro modulated macrophages from wild type and TLR2-deficient mice demonstrated that exposure of macrophages to *Wolbachia* or LsAg improved *E. coli*-induced sepsis in a TLR2-dependent manner. Similarly, transfer of macrophages from *L. sigmodontis*-infected mice improved sepsis outcome more efficiently, than macrophages derived from naïve wild type or *L. sigmodontis*-infected TLR2-deficient mice.

Relevance of endotoxin tolerance and TLR hyporesponsiveness by TLR2-specific ligands have been previously shown in sepsis models and bacterial challenge infections [58–64]. Cellular functions modulated by certain helminth-derived antigens in a TLR2-dependent manner have been demonstrated to manipulate concurrent immune responses and bystander antigens [65–68].

Indeed, several helminth-derived excretory/secretory products have been described to modulate macrophage function directly, preventing TLR activation and improving endotoxemia [16, 28, 30, 69, 70].

Although human data on the impact of filarial infection on sepsis is limited, there are indications that findings from our study can be translated to human filarial infections. For example, it was reported that monocytes from filariae-infected individuals have a significantly reduced TLR2 and TLR4 activation status on the PCR level compared to monocytes derived from uninfected controls [71]. Similarly, Arndts et al. reported that PBMCs from microfilariae-positive *Wuchereria bancrofti*-infected individuals produce less IL-6, TNFα and IL-10 following LPS stimulation [72] and Sasisekhar et al. demonstrated that monocytes of microfilaremic *Wuchereria bancrofti* patients produce less IL-1β after LPS stimulation compared to monocytes from endemic controls [73]. A reduced production of pro-inflammatory cytokines from monocytes of filariasis patients in response to LPS was further reported by Panda et al. and correlated with the upregulation of alternative activation markers [28]. The authors of this study proposed that circulating filarial antigens (CFA) saturate TLR4 binding which reduces LPS-induced monocyte activation. While a similar mechanism may occur in chronically infected *L. sigmodontis* mice, our transfer experiments using in vitro modulated macrophages suggest that saturation of TLR4 is not the mode of action in our study. The only human study that investigated the impact of filarial infection on sepsis in human subjects yet further highlights that filarial infection may indeed have beneficial effects on sepsis. There, Panda et al. reported that significantly less sepsis patients were CFA positive in comparison to healthy endemic controls [17].

We conclude that chronic filarial infections help maintain a balanced immune system during sepsis by functionally modulating macrophages that prevents excessive inflammation and enhances anti-bacterial functions. As the human immune system evolved in the presence of parasitic infections, loss of helminth infections due to improved hygiene may lead to an unbalanced immune system that does not only favor the development of autoimmune diseases but may also promote exacerbated pro-inflammatory immune responses during bacterial infections. Therefore, tolerogenic treatment strategies that employ immunomodulatory molecules derived from parasitic helminths or transfer of in vitro modulated cells that prevent exacerbated inflammation without impairing bacterial clearance may present new therapeutic avenues to improve sepsis survival in humans.

## Materials and Methods

### Ethics statement

Animal housing conditions and the procedures used in this work were performed according to the European Union animal welfare guidelines. All protocols were approved by the Landesamt für Natur, Umwelt und Verbraucherschutz, Cologne, Germany (AZ 87–51.04.2010.A066 and 84–02.04.2011.A326).

### Mice and parasites

All wild type mice were purchased from Janvier Labs, Saint-Berthevin, France. Gene deficient BALB/c mice were kindly provided by Prof. Dr. Klaus Matthaei, Australia National University College of Medicine, Biology and Environment, Canberra, Australia, (IL-4Rα/IL-5^-/-^ mice), Prof. Dr. Frank Brombacher, International Centre for Genetic Engineering and Biotechnology, Cape Town, South Africa (IL-4^-/-^ mice) and the CNRS University of Orléans, France (TLR2^-/-^ and TLR4^-/-^ mice) and were bred and housed at animal facilities of the University Hospital of Bonn. Mice were kept in individually ventilated cages with access to food and water ad libitum.

Six to eight week old, female BALB/c, TLR2^-/-^, IL-4Rα/IL-5^-/-^ and IL-4^-/-^ mice were infected with *L. sigmodontis* by natural infection as described before [74]. Ninety days post infection, a time point of chronic infection, experiments were performed. After euthanasia, infection of mice was confirmed by screening for adult worms in the thoracic cavity and microfilariae in the peripheral blood.

### Sepsis induction

For sepsis induction, mice were i.p. injected with 2–20 × 10^7^ cfu of *E. coli* (ATTC 25922). Body temperature was determined hourly by infra-red measurement. Six hours after injection mice were euthanized and blood and peritoneal lavage were taken for ELISA and flow cytometric analysis as well as cfu determination. For mock treatment, control animals received i.p. injections of 200µl of sterile lysogeny *broth* (LB medium). For sepsis survival experiments, 0.5–1 × 10^9^ cfu were injected and mice were monitored for signs of convulsion, paralysis and low body temperature (<27°C). Mice showing these severe symptoms do not survive sepsis and were therefore euthanized according to humane endpoint criteria.

### Determination of cytokine, chemokine and nitrite concentrations and cfu

Six hours after *E. coli* challenge mice were euthanized and the peritoneum of mice were lavaged with 5ml of cold PBS (PAA, Cölbe, Germany). Following centrifugation of the lavage, the supernatant was stored at -20°C for cytokine, chemokine and nitrite measurement at a later time point. Part of the peritoneal lavage was plated in serial dilutions on LB agar plates and incubated overnight at 37°C to determine the cfu. Peritoneal cells were prepared for subsequent analysis as described below.

To determine cytokine and chemokine concentrations in serum and cell culture supernatants, ELISAs were performed according to kit protocols (TNFα, IL-1β: eBioscience, San Diego, USA; IL-6, IL-10: BD Biosciences, San Diego, USA; MIP-2β, KC/CXCL1 and IL-5: R&D systems, Minneapolis, USA). To determine nitrite concentrations in supernatants, the Griess reagent assay was performed according to the kit protocol (Thermo Fisher Scientific, Waltham, USA). Data was acquired using a microplate reader and Softmax Pro software (both Molecular Devices, Sunnyvale, USA).

### Flow cytometry

For flow cytometric analysis, cells were fixed in fixation/permeabilization buffer (eBioscience) overnight, washed and blocked in PBS containing 1% bovine serum albumin (BSA) and rat immunoglobulin (1µg/ml, Sigma, St. Louis, USA). Cells were stained with F4/80 APC or PerCP-Cy5.5, CD11b APC or FITC, Gr1 PE-Cy7, CD80 FITC, CD86 PE or APC, MHC-II PE or FITC, CD40 PE, Ly6C PerCP-Cy5.5 (all eBioscience) and SiglecF PE (BD Biosciences). To stain for RELMα-positivity, cells were pre-incubated in permeabilization buffer (eBioscience) and then stained with anti-RELMα (Peprotech, New Jersey, USA). Subsequently, cells were washed twice in permeabilization buffer and a secondary antibody (goat anti-rabbit Alexa488, Invitrogen, Carlsbad, USA) was used. As a control unspecific and isotope identical Alexa488 antibody (Invitrogen) was used. Data was acquired using a BD FACS Canto and BD FACSDiva software; for generation of figures and plots FlowJo software (Tree Star, Ashland, USA) was used.

### Macrophage depletion with clodronate liposomes

Clodronate containing liposomes and PBS containing liposomes as a negative control were kindly provided by N. Van Rooijen (Clodronate Liposomes Foundation, The Netherlands; http://clodronate.liposomes.com) and used in our experiments to deplete macrophages in vivo [75] from filariae-infected mice and controls prior to sepsis induction. Therefore, mice were i.p. injected with 100µl of sterile liposome suspension on day -3 and -1 before the mice were challenged i.p. with *E. coli*. Successful depletion of macrophages was confirmed by flow cytometric analyses of the peritoneal lavage and peripheral blood.

### Macrophage elicitation and stimulation

Thioglycollate elicited macrophages were isolated by peritoneal lavage four days after naïve BALB/c mice were i.p. injected with sterile thioglycollate broth. Equal numbers of peritoneal cells were allowed to adhere to cell culture dishes for two hours. After that, non-adhered cells were removed and adherent cells were washed twice resulting in a macrophage purity based on F4/80 positivity of >95%. Macrophages were cultured in RPMI 1640 medium containing 10% fetal calf serum (heat inactivated), 1% Penicillin/Streptomycin and 1% L-Glutamine (all from PAA) and stimulated for a total of 18h. For stimulation, LPS ultrapure (300ng/ml), Pam_3_CSK4 (P_3_C, 100ng/ml), FSL-1 (100ng/ml) were used (all Invivogen, San Diego, USA). *L. sigmodontis* adult worm extract (LsAg) and *L. sigmodontis* adult worm extract from *Wolbachia*-depleted adult worms (Ls-tet) were prepared as previously described [76, 77] and used at a concentration of 25µg/ml for stimulation. Extracts from the insect cell line C6/36 were used at a concentration of 6µg/ml for both control and *Wolbachia*-infected insect cells. For re-stimulation experiments, cells were initially stimulated for 18h as described above, then washed twice and re-stimulated using LPS ultrapure (300ng/ml) or medium for an additional 18 hours. Subsequently, supernatants were collected for cytokine/chemokine determination; cells were washed and detached with a cell scraper, blocked and stained for flow cytometric analyses. LsAg was tested for endotoxin (LPS)-contamination in the Limulus amebocyte lysate (LAL) test (QCL-1000 Test, Lonza, Cologne, Germany), revealing a LPS concentration of 18 pg/ml or 0.18 EU/ml (endotoxin concentration in culture: 0.45 pg/ml).

### Gentamycin assay for in vivo phagocytosis assessment

2×10^7^ cfu *E. coli* (ATTC 25922) were injected i.p. into d90 *L. sigmodontis*-infected BALB/c mice and naïve controls. Three hours after inoculation, mice were killed and cells were obtained by peritoneal lavage. Equal numbers of macrophages were allowed to adhere to cell culture dishes for two hours at 37°C in RPMI 1640 medium containing gentamycin (100µg/ml, PAA). Non-adherent cells were removed and adherent cells were cultured for an additional four hours in gentamycin medium (100µg/ml). Subsequently, adherent macrophages were lysed in 1% Triton-X100 and lysates were plated on LB agar and incubated overnight.

### Phagocytosis of pHrodo-*E. coli* and *S. aureus* BioParticles

Chronic *L. sigmodontis*-infected mice and uninfected controls were i.p. injected with 100µg of pHrodo-*E. coli* or *S. aureus* BioParticles from Thermo Scientific. At 90 min post injection (*E. coli*) or 3h post injection (*S. aureus*), mice were euthanized and peritoneal lavage was analyzed by flow cytometry to assess the frequencies of pHrodo positive macrophages.

### Macrophage gene expression analysis

Three hours after i.p. *E. coli* injection mice were euthanized and peritoneal cells were obtained. Additional controls included peritoneal cells from *L. sigmodontis*-infected and naïve mice in the absence of an *E. coli* challenge. Peritoneal cells were washed in PBS and incubated in supplemented RPMI 1640 medium for one hour for separation by adhesion. Non-adherent cells were removed and adherent cells were stained with F4/80-biotin after blocking in PBS containing 1% BSA and rat Ig (1µg/ml). F4/80-positive macrophages were further purified by magnetic separation using Streptavidin coated magnetic beads (MACS, Miltenyi Biotech, Bergisch-Gladbach, Germany) resulting in a purity of >95%.

RNA was isolated from purified macrophages using Trizol extraction (Ambion, Austin, USA) and the RNeasy Plus Mini Kit (Qiagen, Hilden, Germany). RNA was quantified by NanoVue (GE Lifescience, Chalfont St Giles, Great Britain) and quality was assessed using the Experion gel electrophoresis system (Bio-Rad, Hercules, USA). cDNA was synthesized with the RT2 first strand kit (Qiagen). A customized RT2 PCR array (Qiagen) was performed on cDNA using a Rotor Gene Q (Qiagen). The complete list of genes included in this array is reported in S1 table. Three biological replicates per group were performed. Data was processed and displayed using the online RT2 Profiler PCR Array Data analysis 3.5 software at the sabiociences.com website (Qiagen). Gene expression was normalized to 5 housekeeping genes (Actb, B2m, Gapdh, Gusb, Hsp90ab1).

### In vivo treatment with helminth antigens and TLR agonists

Chronic *L. sigmodontis*-infected BALB/c mice and uninfected controls were i.p. injected every fourth day for a total of three injections with 40µg of LsAg, Ls-tet, *Wolbachia*-infected insect cells, or Pam_3_Cys. Controls received mock injections with PBS. Two days after the last injection mice were challenged with an i.p. injection of 4.5 × 10^8^ *E. coli* bacteria.

### Macrophage transfer experiments

For macrophage transfer experiments using in vitro modulated macrophages, macrophages were obtained from thioglycollate-injected naïve wild type and TLR2-deficient mice and purified by adhesion for 1.5h before in vitro stimulation with 25µg/ml LsAg, *Wolbachia*-infected insect cells or medium as control for 24h. 2.5 million macrophages were then i.p. injected into naïve wild type mice. The purity of F4/80-positive macrophages used for transfer exceeded 85% as determined by flow cytometry.

In a separate experiment, peritoneal macrophages were isolated from chronic *L. sigmodonitis*-infected wild type and TLR2-deficient mice 90 dpi and uninfected wild type controls. After purification of peritoneal macrophages by adhesion for 1.5h, 2.5 million macrophages were transferred in naïve wild type animals by i.p. injection. The purity of F4/80-positive macrophages used for transfer exceeded 85% as determined by flow cytometry. Sepsis was induced in both experiments by i.p. *E. coli* injection (4–5×10^8^ *E. coli* bacteria) 12h after macrophage transfer.

### Statistics

GraphPad Prism software Version 5.03 (GraphPad Software, San Diego, USA) was used for statistical analysis. Mann-Whitney-U-test tested differences between two unpaired groups for significance. Differences between multiple groups were tested for significance using the Kruskal–Wallis test, followed by Dunn post hoc multiple comparisons. Differences between multiple groups over time were analyzed using 2-way ANOVA and Bonferroni post hoc test. Survival data were analyzed for statistical significance using Mantel-Cox Log-rank test. P-values of <0.05 were considered statistically significant. The statistics from the PCR array data were analyzed according to the software provided by SABioscience (Frederick, USA). The p values were hereby calculated based on a Student’s t-test of the replicate 2^(- Delta Ct) values for each gene in the control group and treatment groups, and p values less than 0.05 were considered as significant.

## Supporting Information

S1 Fig Peritoneal macrophages from *L. sigmodontis*-infected BALB/c mice express higher levels of RELMα only after *E. coli* challenge. (**A**) Gating strategy to identify RELMα-positive macrophages in *L. sigmodontis* (*L.s.*) and uninfected animals six hours after *E. coli* challenge. Macrophages were gated based on SSC-A and F4/80-positivity and subsequently displayed for their expression of RELMα. (**B**) Mean fluorescence intensity of RELMα on F4/80-positive peritoneal macrophages from *L.s.*-infected animals 6h following mock or *E. coli* treatment.
(TIF)

S2 Fig Peritoneal monocyte frequencies, but not neutrophil numbers are reduced following clodronate-liposome treatment in *E. coli*-challenged mice. (**A**) Frequency of F4/80^hi^ CD11b^hi^ macrophages, (**B**) Ly6C^+^ F4/80^neg^ monocytes and (**C**) total number of Gr1^+^ F4/80^neg^ neutrophils in the peritoneum after clodronate-Iiposome treatment and *E. coli* injection. Graphs show data obtained 6 hours after *E. coli* challenge or mock treatment. Data is presented as median and was tested for statistical significance by 1-way ANOVA followed by Dunn’s multiple comparisons test.*p< 0.05; **p< 0.01; ***p< 0.001.
(TIF)

S3 Fig Neutrophil recruitment to the peritoneum is not affected in AAM-deficient IL-4Rα/IL-5^-/-^ mice. Total number of peritoneal Gr1^+^, F4/80^neg^ peritoneal neutrophils six hours after *E. coli* injection in *L. sigmodontis* (*L.s.*)-infected and uninfected wild type and IL-4Rα/IL-5-deficient BALB/c mice. Data is presented as median and was tested for statistical significance by 1-way ANOVA followed by Dunn’s multiple comparisons test. **p< 0.01.
(TIF)

S4 Fig *L. sigmodontis*-mediated protection against *E. coli*-induced sepsis is not compromised in AAM-deficient IL-4^-/-^ mice. (**A**) Kinetic of body temperature in response to i.p. *E. coli* injection in uninfected (U) and chronic *L. sigmodontis* (*L.s.*)-infected wild type and IL-4-deficient BALB/c mice. (**B**) Peritoneal bacterial load six hours post *E. coli* injection. Data shown in (**A**) is displayed as mean +/- SEM and was tested for statistical significance by 2-way ANOVA and Bonferroni post-hoc test (asterisks indicate significant differences between *L.s.*-infected and uninfected IL-4^-/-^ mice and paragraphs between *L.s.*-infected and uninfected wild type mice. Data in (**B**) is presented as median and was tested for statistical significance by 1-way ANOVA followed by Dunn’s post-hoc test. *p<0.05; **p<0.01, ***p<0.001.
(TIF)

S5 Fig Macrophage stimulation with *Wolbachia*-containing preparations of *L. sigmodontis* adult worms and insect cells induce TLR2-dependent tolerance to subsequent LPS stimulation. Thioglycollate-elicited peritoneal macrophages from wild type and TLR2-deficient mice were stimulated (prime) for 18 hours with TLR4- (*E. coli*-LPS) and TLR2- (P_3_C) specific ligands, *L. sigmodontis* adult worm extract (LsAg), LsAg from *Wolbachia*-depleted worms (Ls-tet), *Wolbachia*-infected (WOLB) and sterile preparations of C6/36 insect cells before re-stimulation with LPS for an additional 18 hours. Mean fluorescence intensity (MFI) of MHC-II (**A**) and CD40 (**B**) on F4/80^hi^ macrophages are plotted relative to the acute LPS response (medium prime/LPS stimulation). Shown are data representative of three independent experiments (n = 3 per group). Bars show mean + SEM and were tested for statistical significance by student’s t-test. Asterisks on top of the bars indicate significant differences compared to med/LPS conditions. *p<0.05; **p<0.01; ***p<0.001.
(TIF)

S1 Table List of genes included in the PCR array analysis. Displayed are fold-changes and p-values of genes expressed in macrophages derived from *L. sigmodontis*-infected, *L. sigmodontis*-infected and *E. coli* challenged as well as *E. coli*-only challenged mice in comparison to macrophage gene expression of naïve mice.
(DOCX)

S2 Table Displayed are fold-changes and p-values of genes expressed in peritoneal macrophages derived from *L. sigmodontis*-infected and *E. coli* challenged mice in comparison to gene expression of macrophages of *E. coli*-only challenged controls. (DOCX)
