# Supplementary material for: Chronic Filarial Infection Provides Protection against Bacterial Sepsis by Functionally Reprogramming Macrophages
Source: PLoS Pathog. 2015 Jan 22;11(1):e1004616. doi: 10.1371/journal.ppat.1004616 (PMC4303312; doi:10.1371/journal.ppat.1004616)
Supplement: S1 Table — Displayed are fold-changes and p-values of genes expressed in macrophages derived from L. sigmodontis-infected, L. sigmodontis-infected and E. coli challenged as well as E. coli-only challenged mice in comparison to macrophage gene expression of naïve mice. (DOCX) [file ppat.1004616.s006.docx]

**Table S1.** List of genes included in the PCR array analysis. Displayed are fold-changes and p-values of genes expressed in macrophages derived from *L. sigmodontis* infected, *L. sigmodontis* infected and *E. coli* challenged as well as *E. coli*-only challenged mice in comparison to macrophage gene expression of naïve mice.

| \|  \| ***L. sigmodontis*** \| \| ***E. coli*** \| \| ***L.s.+ E. coli*** \| \| \| --- \| --- \| --- \| --- \| --- \| --- \| --- \| \| **Symbol** \| **Fold-change** \| **p-value** \| **Fold-change** \| **p-value** \| **Fold-change** \| **p-value** \| \| CCL24, Eotaxin-2 \| 2*.*15 \| 0*.*1463 \| 3*.*93 \| 0*.*0295 \| 2*.*10 \| 0*.*2747 \| \| BTK \| -1*.*42 \| 0*.*2702 \| 1*.*25 \| 0*.*4727 \| 1*.*28 \| 0*.*4845 \| \| C/EBP \| -2*.*37 \| 0*.*0523 \| 7*.*33 \| 0*.*0025 \| 9*.*49 \| 0*.*2522 \| \| IKKa \| 2*.*17 \| 0*.*1944 \| 1*.*35 \| 0*.*4613 \| 1*.*58 \| 0*.*1307 \| \| IKKe \| 1*.*64 \| 0*.*3995 \| 9*.*39 \| 0*.*0028 \| 31*.*33 \| 0*.*2238 \| \| cFos \| 1*.*04 \| 0*.*8569 \| 1*.*48 \| 0*.*5169 \| -1*.*32 \| 0*.*9408 \| \| IRAK-1 \| -1*.*22 \| 0*.*4894 \| 1*.*55 \| 0*.*1036 \| -1*.*00 \| 0*.*8531 \| \| IRF1 \| 1*.*14 \| 0*.*5821 \| 36*.*29 \| 0*.*0120 \| 22*.*25 \| 0*.*2279 \| \| IRF3 \| 1*.*58 \| 0*.*3265 \| 1*.*59 \| 0*.*2095 \| 1*.*31 \| 0*.*4662 \| \| IRF5 \| -2*.*05 \| 0*.*0349 \| 4*.*81 \| 0*.*0763 \| 4*.*83 \| 0*.*0000 \| \| IRF7 \| 1*.*83 \| 0*.*3897 \| 2*.*02 \| 0*.*5265 \| 3*.*46 \| 0*.*2282 \| \| cJun, AP-1 \| -1*.*30 \| 0*.*5233 \| 3*.*50 \| 0*.*0021 \| 2*.*71 \| 0*.*0181 \| \| MyD88 \| -1*.*31 \| 0*.*6983 \| 5*.*42 \| 0*.*0003 \| 5*.*23 \| 0*.*2185 \| \| NFkB, p50, p105 \| -2*.*01 \| 0*.*0842 \| 3*.*96 \| 0*.*0224 \| 3*.*29 \| 0*.*0002 \| \| NFkB, p52, p100 \| -2*.*43 \| 0*.*3858 \| 3*.*87 \| 0*.*0666 \| 6*.*20 \| 0*.*0112 \| \| IkBbeta \| -1*.*94 \| 0*.*0465 \| 8*.*23 \| 0*.*0006 \| 5*.*58 \| 0*.*0238 \| \| IKKb \| -1*.*20 \| 0*.*8819 \| 5*.*97 \| 0*.*0143 \| 3*.*65 \| 0*.*0289 \| \| PPARa \| -1*.*32 \| 0*.*8331 \| 13*.*94 \| 0*.*0628 \| 1*.*84 \| 0*.*3715 \| \| PPARg \| 2*.*76 \| 0*.*3199 \| 16*.*16 \| 0*.*0668 \| 9*.*87 \| 0*.*0012 \| \| NFkB, cRel \| -3*.*46 \| 0*.*0350 \| 1*.*02 \| 0*.*9394 \| -1*.*06 \| 0*.*6713 \| \| NFkB, RelA, p65 \| -3*.*46 \| 0*.*0361 \| 3*.*17 \| 0*.*0340 \| 2*.*03 \| 0*.*0238 \| \| NFkB, RelB \| -2*.*21 \| 0*.*0155 \| -1*.*36 \| 0*.*1398 \| 1*.*69 \| 0*.*1033 \| \| HMGB1 \| 1*.*17 \| 0*.*5135 \| -1*.*85 \| 0*.*0321 \| -2*.*13 \| 0*.*0460 \| \| RICK/RIP2 \| 1*.*29 \| 0*.*2954 \| -1*.*62 \| 0*.*1695 \| -1*.*01 \| 0*.*9562 \| \| TRIF \| -2*.*87 \| 0*.*0179 \| 6*.*94 \| 0*.*0193 \| 5*.*31 \| 0*.*0242 \| \| TRAM \| -1*.*96 \| 0*.*0848 \| -2*.*37 \| 0*.*1391 \| -2*.*89 \| 0*.*0228 \| \| Mal/Tirap \| -1*.*78 \| 0*.*1059 \| 1*.*59 \| 0*.*2380 \| 2*.*71 \| 0*.*3088 \| \| A20/ TNFAIP3 \| -1*.*84 \| 0*.*2331 \| 11*.*09 \| 0*.*0530 \| 12*.*96 \| 0*.*0476 \| \| TOLLIP \| -1*.*71 \| 0*.*1722 \| 5*.*30 \| 0*.*0161 \| 3*.*60 \| 0*.*0316 \| \| TRADD \| 2*.*42 \| 0*.*2310 \| 1*.*65 \| 0*.*2826 \| 1*.*52 \| 0*.*4138 \| \| Traf6 \| -2*.*89 \| 0*.*0120 \| 1*.*58 \| 0*.*1444 \| 1*.*21 \| 0*.*4613 \| \| CD14 \| 2*.*08 \| 0*.*1667 \| -1*.*14 \| 0*.*9811 \| 1*.*58 \| 0*.*4431 \| \| CD80 \| -1*.*64 \| 0*.*2804 \| 4*.*14 \| 0*.*0359 \| 2*.*56 \| 0*.*0518 \| \| CD86 \| -1*.*62 \| 0*.*4151 \| 3*.*41 \| 0*.*0334 \| 1*.*27 \| 0*.*5688 \| \| MD2 \| 1*.*71 \| 0*.*3922 \| 1*.*40 \| 0*.*6532 \| 2*.*89 \| 0*.*2378 \| \| TLR1 \| 1*.*21 \| 0*.*5248 \| -2*.*04 \| 0*.*0571 \| 1*.*20 \| 0*.*4770 \| \| TLR2 \| -1*.*36 \| 0*.*4235 \| 1*.*36 \| 0*.*3549 \| 3*.*12 \| 0*.*2475 \| \| IRAK-4 \| 1*.*39 \| 0*.*2166 \| 1*.*04 \| 0*.*8122 \| 1*.*24 \| 0*.*5462 \| \| TLR4 \| 1*.*38 \| 0*.*6640 \| 1*.*88 \| 0*.*3178 \| 3*.*38 \| 0*.*2733 \| \| TLR6 \| -1*.*53 \| 0*.*2733 \| -2*.*83 \| 0*.*2196 \| 1*.*26 \| 0*.*3901 \| \| TNFaR \| 1*.*19 \| 0*.*7069 \| 2*.*12 \| 0*.*2250 \| 3*.*19 \| 0*.*3451 \| \| CD40 \| 1*.*03 \| 0*.*7069 \| 4*.*14 \| 0*.*0001 \| 3*.*07 \| 0*.*0600 \| \| CCR2 \| 1*.*01 \| 0*.*7116 \| 2*.*79 \| 0*.*1925 \| 2*.*87 \| 0*.*2264 \| \| CCR5 \| 1*.*20 \| 0*.*7101 \| -2*.*79 \| 0*.*1453 \| -1*.*15 \| 0*.*8073 \| \| IRAK-M \| -1*.*19 \| 0*.*4889 \| 1*.*99 \| 0*.*0492 \| 4*.*42 \| 0*.*2185 \| \| Leptin Receptor \| 14*.*42 \| 0*.*0884 \| 23*.*77 \| 0*.*0690 \| 9*.*66 \| 0*.*2899 \| \| IFN (alpha,beta) receptor 1 \| -1*.*52 \| 0*.*7015 \| 4*.*34 \| 0*.*0040 \| 6*.*47 \| 0*.*1137 \| \| CCL22 \| -9*.*54 \| 0*.*1551 \| 1*.*69 \| 0*.*5060 \| 1*.*37 \| 0*.*8470 \| \| IL1RA \| 1*.*59 \| 0*.*4865 \| 41*.*39 \| 0*.*0455 \| 59*.*82 \| 0*.*1496 \| \| M-CSF \| -1*.*47 \| 0*.*5385 \| 32*.*18 \| 0*.*0167 \| 12*.*63 \| 0*.*1843 \| \| GM-CSF \| -1*.*07 \| 0*.*9923 \| 14*.*20 \| 0*.*0190 \| 5*.*68 \| 0*.*3558 \| \| CXCL10, IP-10 \| 5*.*72 \| 0*.*3771 \| 545*.*46 \| 0*.*0340 \| 276*.*79 \| 0*.*3337 \| \| IFNb \| 5*.*53 \| 0*.*3612 \| 47*.*88 \| 0*.*0543 \| 8*.*22 \| 0*.*0741 \| \| IFNg \| -1*.*29 \| 0*.*5093 \| 39*.*52 \| 0*.*0492 \| 24*.*30 \| 0*.*3726 \| \| C5aR \| 1*.*43 \| 0*.*3372 \| 4*.*29 \| 0*.*0945 \| 11*.*05 \| 0*.*2639 \| \| CCL2/MCP1 \| 1*.*93 \| 0*.*4045 \| -4*.*24 \| 0*.*0224 \| -2*.*49 \| 0*.*3281 \| \| TNFa \| -2*.*68 \| 0*.*0904 \| 17*.*85 \| 0*.*0407 \| 26*.*77 \| 0*.*1463 \| \| IL10 \| -9*.*54 \| 0*.*1504 \| 9*.*59 \| 0*.*0081 \| 7*.*03 \| 0*.*0244 \| \| IL12p35 \| -1*.*53 \| 0*.*3970 \| 6*.*00 \| 0*.*0044 \| 6*.*88 \| 0*.*1671 \| \| IL1b \| 1*.*82 \| 0*.*3321 \| 5*.*64 \| 0*.*1440 \| 10*.*10 \| 0*.*2639 \| \| IL6 \| -5*.*72 \| 0*.*1656 \| -11*.*38 \| 0*.*1249 \| -8*.*21 \| 0*.*1563 \| \| Socs1 \| -2*.*29 \| 0*.*2154 \| 38*.*53 \| 0*.*0281 \| 31*.*76 \| 0*.*1644 \| \| Socs3 \| -1*.*82 \| 0*.*1754 \| 3*.*02 \| 0*.*0432 \| 7*.*36 \| 0*.*2705 \| \| CD11b/ CR3/Mac-1 \| 1*.*47 \| 0*.*7668 \| 2*.*69 \| 0*.*0935 \| 2*.*65 \| 0*.*3753 \| \| SHIP-1 \| -1*.*11 \| 0*.*8178 \| 9*.*39 \| 0*.*0165 \| 4*.*95 \| 0*.*0016 \| \| MBL \| 2*.*43 \| 0*.*4023 \| 114*.*40 \| 0*.*0527 \| 11*.*95 \| 0*.*2039 \| \| NOD2 \| 1*.*55 \| 0*.*4490 \| 22*.*75 \| 0*.*0002 \| 28*.*23 \| 0*.*1958 \| \| C3 \| 1*.*69 \| 0*.*3609 \| 4*.*64 \| 0*.*0029 \| 11*.*73 \| 0*.*2479 \| \| iNOS \| 1*.*24 \| 0*.*4051 \| 56*.*94 \| 0*.*0196 \| 271*.*72 \| 0*.*3338 \| \| COX2 \| -2*.*29 \| 0*.*2580 \| 4*.*73 \| 0*.*2833 \| 5*.*07 \| 0*.*2584 \| \| IL1R \| -2*.*85 \| 0*.*6434 \| 2*.*88 \| 0*.*1309 \| 2*.*32 \| 0*.*2584 \| \| IL6R \| -1*.*22 \| 0*.*6691 \| 2*.*56 \| 0*.*1144 \| 5*.*11 \| 0*.*0045 \| \| YM1 \| 2*.*68 \| 0*.*2912 \| 212*.*50 \| 0*.*0013 \| 1280*.*66 \| 0*.*1127 \| \| AMCase \| 1*.*68 \| 0*.*7528 \| 18*.*23 \| 0*.*0654 \| 2*.*76 \| 0*.*3749 \| \| MR \| 6*.*70 \| 0*.*0181 \| 12*.*86 \| 0*.*0545 \| 5*.*46 \| 0*.*0076 \| \| RELMa \| 6*.*59 \| 0*.*3196 \| 36560*.*55 \| 0*.*0089 \| 10174*.*47 \| 0*.*2411 \| \| IL4 \| -1*.*31 \| 0*.*7967 \| 77*.*78 \| 0*.*0013 \| 5*.*16 \| 0*.*0750 \| \| IL4Ra \| -1*.*44 \| 0*.*7776 \| 4*.*29 \| 0*.*0094 \| 6*.*95 \| 0*.*1525 \| \| Cx3CR1 \| -1*.*11 \| 0*.*9211 \| 19*.*49 \| 0*.*0638 \| 3*.*10 \| 0*.*2559 \| \| IL13 \| 1*.*23 \| 0*.*5616 \| 51*.*55 \| 0*.*0996 \| 8*.*73 \| 0*.*0483 \| \| ST2 \| -2*.*70 \| 0*.*2864 \| 2*.*07 \| 0*.*2673 \| -4*.*40 \| 0*.*2100 \| \| IL33 \| -1*.*12 \| 0*.*4654 \| 12*.*62 \| 0*.*0725 \| 8*.*75 \| 0*.*2670 \| \| TGFb \| -1*.*09 \| 0*.*9970 \| 4*.*14 \| 0*.*0010 \| 2*.*30 \| 0*.*0407 \| \| CD273/PD-L2/B7-DC \| -1*.*63 \| 0*.*5369 \| 8*.*44 \| 0*.*0032 \| 2*.*96 \| 0*.*1576 \| \| Arginase 1 \| 3*.*85 \| 0*.*3820 \| -7*.*35 \| 0*.*2365 \| -1*.*96 \| 0*.*6440 \| \| F4/80 \| 2*.*64 \| 0*.*0499 \| -1*.*47 \| 0*.*2654 \| -4*.*86 \| 0*.*0218 \| \| PPARd \| -1*.*38 \| 0*.*0196 \| 1*.*24 \| 0*.*4139 \| 2*.*51 \| 0*.*0739 \| \| CCL8 /MCP2 \| 105*.*18 \| 0*.*3074 \| 114*.*40 \| 0*.*0527 \| 26*.*10 \| 0*.*2928 \| |
| --- | --- | --- | --- | --- | --- | --- | --- | --- | --- | --- | --- | --- | --- | --- | --- | --- | --- | --- | --- | --- | --- | --- | --- | --- | --- | --- | --- | --- | --- | --- | --- | --- | --- | --- | --- | --- | --- | --- | --- | --- | --- | --- | --- | --- | --- | --- | --- | --- | --- | --- | --- | --- | --- | --- | --- | --- | --- | --- | --- | --- | --- | --- | --- | --- | --- | --- | --- | --- | --- | --- | --- | --- | --- | --- | --- | --- | --- | --- | --- | --- | --- | --- | --- | --- | --- | --- | --- | --- | --- | --- | --- | --- | --- | --- | --- | --- | --- | --- | --- | --- | --- | --- | --- | --- | --- | --- | --- | --- | --- | --- | --- | --- | --- | --- | --- | --- | --- | --- | --- | --- | --- | --- | --- | --- | --- | --- | --- | --- | --- | --- | --- | --- | --- | --- | --- | --- | --- | --- | --- | --- | --- | --- | --- | --- | --- | --- | --- | --- | --- | --- | --- | --- | --- | --- | --- | --- | --- | --- | --- | --- | --- | --- | --- | --- | --- | --- | --- | --- | --- | --- | --- | --- | --- | --- | --- | --- | --- | --- | --- | --- | --- | --- | --- | --- | --- | --- | --- | --- | --- | --- | --- | --- | --- | --- | --- | --- | --- | --- | --- | --- | --- | --- | --- | --- | --- | --- | --- | --- | --- | --- | --- | --- | --- | --- | --- | --- | --- | --- | --- | --- | --- | --- | --- | --- | --- | --- | --- | --- | --- | --- | --- | --- | --- | --- | --- | --- | --- | --- | --- | --- | --- | --- | --- | --- | --- | --- | --- | --- | --- | --- | --- | --- | --- | --- | --- | --- | --- | --- | --- | --- | --- | --- | --- | --- | --- | --- | --- | --- | --- | --- | --- | --- | --- | --- | --- | --- | --- | --- | --- | --- | --- | --- | --- | --- | --- | --- | --- | --- | --- | --- | --- | --- | --- | --- | --- | --- | --- | --- | --- | --- | --- | --- | --- | --- | --- | --- | --- | --- | --- | --- | --- | --- | --- | --- | --- | --- | --- | --- | --- | --- | --- | --- | --- | --- | --- | --- | --- | --- | --- | --- | --- | --- | --- | --- | --- | --- | --- | --- | --- | --- | --- | --- | --- | --- | --- | --- | --- | --- | --- | --- | --- | --- | --- | --- | --- | --- | --- | --- | --- | --- | --- | --- | --- | --- | --- | --- | --- | --- | --- | --- | --- | --- | --- | --- | --- | --- | --- | --- | --- | --- | --- | --- | --- | --- | --- | --- | --- | --- | --- | --- | --- | --- | --- | --- | --- | --- | --- | --- | --- | --- | --- | --- | --- | --- | --- | --- | --- | --- | --- | --- | --- | --- | --- | --- | --- | --- | --- | --- | --- | --- | --- | --- | --- | --- | --- | --- | --- | --- | --- | --- | --- | --- | --- | --- | --- | --- | --- | --- | --- | --- | --- | --- | --- | --- | --- | --- | --- | --- | --- | --- | --- | --- | --- | --- | --- | --- | --- | --- | --- | --- | --- | --- | --- | --- | --- | --- | --- | --- | --- | --- | --- | --- | --- | --- | --- | --- | --- | --- | --- | --- | --- | --- | --- | --- | --- | --- | --- | --- | --- | --- | --- | --- | --- | --- | --- | --- | --- | --- | --- | --- | --- | --- | --- | --- | --- | --- | --- | --- | --- | --- | --- | --- | --- | --- | --- | --- | --- | --- | --- | --- | --- | --- | --- | --- | --- | --- | --- | --- | --- | --- | --- | --- | --- | --- | --- | --- | --- | --- | --- | --- | --- | --- | --- | --- | --- | --- | --- | --- | --- | --- | --- | --- | --- | --- | --- | --- | --- | --- | --- | --- | --- | --- | --- | --- | --- | --- | --- | --- | --- | --- | --- | --- | --- | --- | --- | --- | --- | --- | --- | --- | --- | --- | --- | --- | --- | --- | --- | --- | --- | --- | --- | --- | --- | --- | --- | --- | --- | --- | --- | --- | --- | --- | --- | --- | --- | --- | --- | --- | --- | --- | --- | --- | --- | --- | --- | --- | --- | --- | --- | --- | --- | --- | --- | --- | --- | --- | --- | --- | --- | --- |
