# Supplementary material for: Chronic Filarial Infection Provides Protection against Bacterial Sepsis by Functionally Reprogramming Macrophages
Source: PLoS Pathog. 2015 Jan 22;11(1):e1004616. doi: 10.1371/journal.ppat.1004616 (PMC4303312; doi:10.1371/journal.ppat.1004616)
Supplement: S2 Table — (DOCX) [file ppat.1004616.s007.docx]

**Table S2.** Displayed are fold-changes and p-values of genes expressed in peritoneal macrophages derived from *L. sigmodontis* infected and *E. coli* challenged mice in comparison to gene expression of macrophages of *E. coli*-only challenged controls.

| \| **Symbol** \| **Fold-change** \| **p-value** \| \| --- \| --- \| --- \| \| CCL24, Eotaxin-2 \| -1*.*87 \| 0*.*3970 \| \| BTK \| 1*.*03 \| 0*.*7505 \| \| C/EBP \| 1*.*29 \| 0*.*4904 \| \| IKKa \| 1*.*17 \| 0*.*7794 \| \| IKKe \| 3*.*34 \| 0*.*2991 \| \| cFos \| -1*.*96 \| 0*.*7909 \| \| IRAK-1 \| -1*.*56 \| 0*.*2596 \| \| IRF1 \| -1*.*63 \| 0*.*9447 \| \| IRF3 \| -1*.*21 \| 0*.*4966 \| \| IRF5 \| 1*.*01 \| 0*.*7846 \| \| IRF7 \| 1*.*72 \| 0*.*3022 \| \| cJun, AP-1 \| -1*.*29 \| 0*.*2441 \| \| MyD88 \| -1*.*04 \| 0*.*6765 \| \| NFkB, p50, p105 \| -1*.*20 \| 0*.*3840 \| \| NFkB, p52, p100 \| 1*.*60 \| 0*.*3077 \| \| IkBbeta \| -1*.*48 \| 0*.*1890 \| \| IKKb \| -1*.*64 \| 0*.*1260 \| \| PPARa \| -7*.*58 \| 0*.*0826 \| \| PPARg \| -1*.*64 \| 0*.*2789 \| \| NFkB, cRel \| -1*.*08 \| 0*.*5760 \| \| NFkB, RelA, p65 \| -1*.*56 \| 0*.*1379 \| \| NFkB, RelB \| 2*.*31 \| 0*.*0496 \| \| HMGB1 \| -1*.*16 \| 0*.*8405 \| \| RICK/RIP2 \| 1*.*60 \| 0*.*2228 \| \| TRIF \| -1*.*31 \| 0*.*4763 \| \| TRAM \| -1*.*22 \| 0*.*4506 \| \| Mal/Tirap \| 1*.*71 \| 0*.*4236 \| \| A20/ TNFAIP3 \| 1*.*17 \| 0*.*7641 \| \| TOLLIP \| -1*.*47 \| 0*.*2737 \| \| TRADD \| -1*.*09 \| 0*.*7764 \| \| Traf6 \| -1*.*31 \| 0*.*3759 \| \| CD14 \| 1*.*80 \| 0*.*4583 \| \| CD80 \| -1*.*62 \| 0*.*2358 \| \| CD86 \| -2*.*69 \| 0*.*1112 \| \| MD2 \| 2*.*06 \| 0*.*2784 \| \| TLR1 \| 2*.*45 \| 0*.*0256 \| \| TLR2 \| 2*.*29 \| 0*.*2855 \| \| IRAK-4 \| 1*.*20 \| 0*.*6644 \| \| TLR4 \| 1*.*80 \| 0*.*6172 \| \| TLR6 \| 3*.*58 \| 0*.*1051 \| \| TNFaR \| 1*.*51 \| 0*.*4581 \| \| CD40 \| -1*.*35 \| 0*.*4398 \| \| CCR2 \| 1*.*03 \| 0*.*7549 \| \| CCR5 \| 2*.*43 \| 0*.*1708 \| | \| **Symbol** \| **Fold-change** \| **p-value** \| \| --- \| --- \| --- \| \| IRAK-M \| 2*.*22 \| 0*.*3109 \| \| Leptin Receptor \| -2*.*46 \| 0*.*5738 \| \| IFN (α,β) receptor 1 \| 1*.*49 \| 0*.*3794 \| \| CCL22 \| -1*.*23 \| 0*.*5233 \| \| IL1RA \| 1*.*45 \| 0*.*5064 \| \| M-CSF \| -2*.*55 \| 0*.*3065 \| \| GM-CSF \| -2*.*50 \| 0*.*9510 \| \| CXCL10, IP-10 \| -1*.*97 \| 0*.*7086 \| \| IFNb \| -5*.*82 \| 0*.*0908 \| \| IFNg \| -1*.*63 \| 0*.*4051 \| \| C5aR \| 2*.*58 \| 0*.*3609 \| \| CCL2/MCP1 \| 1*.*70 \| 0*.*3951 \| \| TNFa \| 1*.*50 \| 0*.*4776 \| \| IL10 \| -1*.*36 \| 0*.*3464 \| \| IL12p35 \| 1*.*15 \| 0*.*5762 \| \| IL1b \| 1*.*79 \| 0*.*4460 \| \| IL6 \| 1*.*39 \| 0*.*5461 \| \| Socs1 \| -1*.*21 \| 0*.*8685 \| \| Socs3 \| 2*.*44 \| 0*.*3552 \| \| CD11b/ CR3/Mac-1 \| -1*.*02 \| 0*.*5880 \| \| SHIP-1 \| -1*.*90 \| 0*.*0913 \| \| MBL \| -9*.*57 \| 0*.*0799 \| \| NOD2 \| 1*.*24 \| 0*.*5083 \| \| C3 \| 2*.*53 \| 0*.*3362 \| \| iNOS \| 4*.*77 \| 0*.*3609 \| \| COX2 \| 1*.*07 \| 0*.*8895 \| \| IL1R \| -1*.*24 \| 0*.*9489 \| \| IL6R \| 2*.*00 \| 0*.*1063 \| \| YM1 \| 6*.*03 \| 0*.*1544 \| \| AMCase \| -6*.*61 \| 0*.*0857 \| \| MR \| -2*.*35 \| 0*.*1426 \| \| RELMa \| -3*.*59 \| 0*.*4036 \| \| IL4 \| -15*.*09 \| 0*.*0018 \| \| IL4Ra \| 1*.*62 \| 0*.*3780 \| \| Cx3CR1 \| -6*.*29 \| 0*.*0929 \| \| IL13 \| -5*.*91 \| 0*.*1438 \| \| ST2 \| -9*.*12 \| 0*.*0906 \| \| IL33 \| -1*.*44 \| 0*.*9023 \| \| TGFb \| -1*.*80 \| 0*.*0354 \| \| CD273/PD-L2/B7-DC \| -2*.*85 \| 0*.*0318 \| \| Arginase 1 \| 3*.*74 \| 0*.*3328 \| \| F4/80 \| -3*.*30 \| 0*.*0638 \| \| PPARd \| 2*.*02 \| 0*.*1530 \| \| CCL8 /MCP2 \| -4*.*38 \| 0*.*3418 \| |
| --- | --- | --- | --- | --- | --- | --- | --- | --- | --- | --- | --- | --- | --- | --- | --- | --- | --- | --- | --- | --- | --- | --- | --- | --- | --- | --- | --- | --- | --- | --- | --- | --- | --- | --- | --- | --- | --- | --- | --- | --- | --- | --- | --- | --- | --- | --- | --- | --- | --- | --- | --- | --- | --- | --- | --- | --- | --- | --- | --- | --- | --- | --- | --- | --- | --- | --- | --- | --- | --- | --- | --- | --- | --- | --- | --- | --- | --- | --- | --- | --- | --- | --- | --- | --- | --- | --- | --- | --- | --- | --- | --- | --- | --- | --- | --- | --- | --- | --- | --- | --- | --- | --- | --- | --- | --- | --- | --- | --- | --- | --- | --- | --- | --- | --- | --- | --- | --- | --- | --- | --- | --- | --- | --- | --- | --- | --- | --- | --- | --- | --- | --- | --- | --- | --- | --- | --- | --- | --- | --- | --- | --- | --- | --- | --- | --- | --- | --- | --- | --- | --- | --- | --- | --- | --- | --- | --- | --- | --- | --- | --- | --- | --- | --- | --- | --- | --- | --- | --- | --- | --- | --- | --- | --- | --- | --- | --- | --- | --- | --- | --- | --- | --- | --- | --- | --- | --- | --- | --- | --- | --- | --- | --- | --- | --- | --- | --- | --- | --- | --- | --- | --- | --- | --- | --- | --- | --- | --- | --- | --- | --- | --- | --- | --- | --- | --- | --- | --- | --- | --- | --- | --- | --- | --- | --- | --- | --- | --- | --- | --- | --- | --- | --- | --- | --- | --- | --- | --- | --- | --- | --- | --- | --- | --- | --- | --- | --- | --- | --- | --- | --- | --- | --- | --- | --- | --- | --- | --- | --- | --- | --- | --- | --- | --- | --- | --- | --- | --- | --- | --- | --- | --- |
